# Comprehensive investigation of a dye-decolorizing peroxidase and a manganese peroxidase from *Irpex lacteus* F17, a lignin-degrading basidiomycete

**DOI:** 10.1186/s13568-018-0648-6

**Published:** 2018-07-17

**Authors:** Zihong Duan, Rui Shen, Binjie Liu, Mengwei Yao, Rong Jia

**Affiliations:** 1 School of Life Science, Economic and Technology Development Zone, Anhui University, 111 Jiulong Road, Hefei, 230601 Anhui People’s Republic of China; 2 Anhui Key Laboratory of Modern Biomanufacturing, Anhui University, Hefei, 230601 People’s Republic of China

**Keywords:** *Irpex lacteus* F17, White-rot fungus, Dye-decolorizing peroxidase, Manganese peroxidase, Redox potential, Biotechnological applications

## Abstract

*Irpex lacteus* F17 is well-known for its ability to degrade recalcitrant aromatic pollutants, which mainly results from the action of the manganese peroxidase (MnP) that it is able to produce. Recently, the genome sequencing and annotation of this strain provided comprehensive picture of the ligninolytic peroxidase gene family. In addition to revealing the presence of 13 MnPs, genes for five dye-decolorizing peroxidases (DyPs) were also discovered in the *I. lacteus* F17 genome, which are unrelated to the fungal class II peroxidases. In the present study, amino acid sequences of five DyPs and 13 MnPs, representing two different families of heme peroxidases, were analyzed. Of these, two enzymes, a DyP (*Il*-DyP4) and a MnP (*Il*-MnP6) were expressed respectively in *Escherichia coli*, and were characterized by comparing their molecular models, substrate specificities, and catalytic features. The results showed that *Il*-DyP4 possessed a higher catalytic efficiency for some representative substrates, and a stronger decolorizing ability to a wide range of synthetic dyes in acidic conditions. Based on electrochemical measurements, *Il*-DyP4 was found to have a high redox potential of 27 mV at pH 3.5, which was superior to that of *Il*-MnP6 (− 75 mV), thereby contributing to its ability to oxidize high redox potential substrates, such as veratryl alcohol and polymeric dye Poly R-478. The results highlighted the potential of *Il*-DyP4 for use in industrial and environmental applications.

**Electronic supplementary material:**

The online version of this article (10.1186/s13568-018-0648-6) contains supplementary material, which is available to authorized users.

## Introduction

Lignocellulose is a structural component of the plant cell wall, mainly comprising cellulose, hemicellulose, and lignin. It is the largest renewable resource in nature and the most promising feedstock for the production of many valuable substances, such as biofuels, chemicals, and materials, from plant sources (Balat 2011). Both cellulose and hemicellulose are carbohydrates, whereas lignin is a highly irregular and insoluble polymer comprising phenylpropanoid units, chemically bonded by mostly ether linkage and carbon–carbon bonds, which are both physically stable and chemically inert (Park et al. 2018). In woody plants, lignin accounts for 20–35% in woody biomass and forms lignin–carbohydrate complexes with cellulose and hemicellulose, resulting in xylem that is extremely hard; however, these complexes are difficult to separate and degrade, thus hindering the conversion and utilization of lignocelluloses (Cheng et al. 2012).

Wood-decaying fungi, one of several groups of lignocellulose decomposers in nature, have an important role in the terrestrial carbon cycle (Kellner et al. 2014). They are common inhabitants of forest litter and fallen trees and have typically been classified as white-rot fungi and brown-rot fungi, according to their ability to biodegrade lignin. Brown-rot fungi are capable of degrading carbohydrates, leaving polyaromatic lignin intact because of their lack of ligninolytic enzymes, whereas white-rot fungi have a ligninolytic system, which can degrade lignin as well as cellulose and hemicelluloses (Morgenstern et al. 2008; Ruiz-Dueñas et al. 2013). Therefore, white-rot fungi have received considerable research because of their extracellular lignolytic enzymes, with a focus on secreted fungal class II peroxidases (PODs), such as lignin peroxidase (LiP; EC 1.11.1.14), manganese peroxidase (MnP; EC 1.11.1.13), and versatile peroxidase (VP; EC 1.11.1.16), as well as other classes of enzyme, such as copper-dependent laccases (Fernández-Fueyo et al. 2014). Thus, two types of fungi can be distinguished by the presence or absence of PODs, which can affect the mode of wood decay adopted by white-rot or brown-rot fungi. However, recently published work, based on the genomic data from 33 species of fungi, suggests that a more nuanced categorization of wood decay modes between white-rot and brown-rot fungi is necessary, because numerous other classes of enzyme were found to be involved in lignin degradation in addition to PODs (Riley et al. 2014). For example, genes encoding PODs are absent in the genomes of *Botryobasidium botryosum* and *Jaapia argillacea* genomes, although both species show modes of lignin degradation that are similar to those of some white-rot fungi. Moreover, the secondary metabolites reducing polyketide synthases (R-PKSs) are abundant in white-rot fungi but reduced in brown-rot fungi. In addition, the dye-decolorizing peroxidases (DyPs), a second new heme peroxidase superfamily, were identified in fungi that have been shown to degrade model lignin compounds, and were also widespread in white-rot fungi, such as *Auricularia delicate* and *Trametes versicolor*, whereas brown-rot fungi lack DyPs (Floudas et al. 2012, 2015).

DyPs are a newly discovered family of heme peroxidases that are unrelated to well-known peroxidases, such as fungal class II PODs, in terms of their amino acid sequence, tertiary structure, and catalytic residues (Colpa et al. 2014). They have a unique protein structure, as well as a distinct amino acid sequence, with a substrate preference for anthraquinone dyes and high peroxidase activity toward a variety of organic compounds (Sugano 2009). The ability of DyPs to oxidize lignin-related compound and nonphenolic lignin model dimers and their low catalytic efficiency on Mn^2+^ and veratryl alcohol have also been reported (Kinne et al. 2011; Colpa et al. 2014; Fernández-Fueyo et al. 2015; Linde et al. 2015; Loncar et al. 2016). All DyPs contain an iron protoporphyrin IX (heme cofactor) as a prosthetic group and the GXXDG motif in their primary sequence as a part of the heme-binding region (Colpa et al. 2014). They also share a conserved spatial structure and a unique ferredoxin-like fold containing α-helices and antiparallel β-sheets (Linde et al. 2015). Nevertheless, the physiological function of these enzymes is unclear, although they exhibit the biotechnological potential (Colpa et al. 2014; van Bloois et al. 2010).

*Irpex lacteus* F17 is an indigenous white-rot basidiomycete isolated by our laboratory that efficiently decolorizes and degrades different synthetic dyes, some of which are recalcitrant for their structural diversity (Zhao et al. 2015; Yang et al. 2016). Initially, this strain was named *Schizophyllum* sp. F17 based on the morphological characteristics of its fruiting body (Cheng et al. 2007) and recognized as *I. lacteus* F17 after identifying the internal transcribed spacer in its nucleotide sequence (GenBank: JQ403610.3.2) and analyzing its phylogenetic tree (Chen et al. 2015). Recently, genome sequencing of *I. lacteus* F17 revealed that the genome encodes 14 Class II PODs and five DyPs. The 14 PODs enzymes include one LiP and 13 putative MnPs. Phylogenetic analysis based on 18S rRNA sequences showed that *I. lacteus* F17 was closely related to *Ceriporiopsis subvermispora* and *Phanerochaete chrysosporium* (Yao et al. 2017). Interestingly, the genome of *C. subvermispora* also encodes 13 MnPs, whereas it has no genes encoding DyPs, similar to *P. chrysosporium* (Fernández-Fueyo et al. 2012; Floudas et al. 2012).

Research has focused mainly on MnPs from *I. lacteus*, probably because of their abundance in the genome of this species (Yao et al. 2017). MnPs from *I. lacteus* are important in the degradation of polycyclic aromatic hydrocarbons and are able to efficiently decolorize different groups of dye (Novotný et al. 2001; Kasinath et al. 2003; Baborová et al. 2006). Recent research indicated that two MnPs from *I. lacteus* CD2 were able to degrade veratryl alcohol, a nonphenolic lignin compound (Qin et al. 2017). The ability of MnP purified from *I. lacteus* F17 to degrade synthetic dyes has also been shown (Chen et al. 2015). However, little is known about the catalytic properties of DyP from *I. lacteus*. To the best of our knowledge, only a wild DyP from *I. lacteus* grown in liquid medium, has been isolated and characterized (Salvachúa et al. 2013). Thus, we selected a DyP gene (No. 8293, GenBank: MG 209114) from the *I. lacteus* F17 genome and expressed it in *Escherichia coli*, and compared it with a MnP gene (No. 6398, GenBank: MG 209112) from the same genome. Any similarities and differences between the two enzymes in terms of their sequence homologies, molecular structures, biochemical and spectroscopic properties, as well as their ability to decolorize different types of dye, were investigated. The results of this study will further reveal the catalytic characteristics of DyPs from *I. lacteus* F17 to provide a better understanding of the lignin degradation potential of this strain.

## Materials and methods

### Regents and expression vectors

Dithiothreitol (DTT), glutathione (GSSG), phenylmethanesulfonyl fluoride (PMSF), and isopropyl β-d-thiogalactopyranoside (IPTG) were from Sigma-Aldrich (St. Louis, MO, USA). Reactive Blue 19 (RBlue 19), reactive black 5 (RBlack 5), veratryl alcohol (VA), 2,6-dimethoxyphenol (DMP), ethylene diamine tetraacetic acid (EDTA), and 2,2-azino-bis(3-ethylbenzothiazoline-6-sulfonic acid) (ABTS) were purchased from Aladdin industrial corporation (Shanghai, China).

The *E. coli* expression vector pET28a(+) and the *E. coli* expression host Rosetta (DE3) were purchased from Invitrogen (Carlsbad, CA, USA) and TransGen Biotech (Beijing, China), respectively.

All restriction enzymes and DNA polymerases were purchased from TaKaRa (Ostu, Japan). Unless otherwise specified, all other reagents used were from Macklin (Shanghai, China) and were of analytical grade.

### Strain and genome

The strain of *I. lacteus* F17 was cultured on potato dextrose agar (PDA) medium (200 g L^−1^ of potato extract, 20 g L^−1^ of glucose, and 20 g L^−1^ of agar) for 5 days at 28 °C and then preserved at 4 °C.

*I. lacteus* F17 was deposited at the China Center for Type Culture Collection (CCTCC) under the accession number of CCTCC AF 2014020. The whole-genome shotgun project has been submitted to GenBank under the accession number MQVO00000000 (http://www.ncbi.nlm.nih.gov).

### DyP and MnP screening of the *I. lacteus* F17 genome

The DyP and MnP coding genes in *I. lacteus* F17 were screened as follows. First, the annotated genome was automatically searched in the NCBI database; second, multiple alignments were performed using Clustal X to compare the deduced amino acid sequences with related peroxidases; finally, the presence of characteristic residues at the heme pocket and substrate oxidation sites were confirmed.

Five DyP genes and 13 MnP genes were identified in the genome of *I. lacteus* F17. The cDNA sequences encoding the 18 mature proteins were submitted to GenBank: *Il*-DyP1 (MH120197), *Il*-DyP2 (MH120198), *Il*-DyP3 (MH120199), *Il*-DyP4 (MG209114), *Il*-DyP5 (MH120196), as well as *Il*-MnP1 (KC811382), *Il*-MnP2 (MH120200), *Il*-MnP3 (MH120201), *Il*-MnP4 (MH120202), *Il*-MnP5 (MH120203), *Il*-MnP6 (MG209112), *Il*-MnP7 (MH120204), *Il*-MnP8 (MH120205), *Il*-MnP9 (MH120206), *Il*-MnP10 (MH120207), *Il*-MnP11 (MH120208), and *Il*-MnP12 (MH120209), *Il*-MnP13 (MH120210).

Based on Protein Blast on NCBI, sequence identity analyses of five DyPs and 13 MnPs in the *I. lacteus* F17 were performed. In addition, homology modeling was performed on the basis of the SWISS MODEL server to obtain the templates of crystal structure with the highest identity in the protein database. The templates were then applied to PyMOL to simulate the three-dimensional structure of the proteins and to superimpose the heme, important amino acids, and ions.

### Gene clone and expression vector construction

Mycelia of *I. lacteus* F17 were cultured in compound potato dextrose agar (CPDA) liquid medium at 28 °C at 120 rpm and harvested on the fourth day. Total RNA was extracted as reported by Koo et al. (1998). The total RNA was then reverse transcribed to cDNA.

*Il*-DyP4 primers DyPf and DyPr (Additional file 1: Table S1) were designed. The above cDNA of *I. lacteus* F17 was used as a template and polymerase chain reactions (PCR) were performed with *Taq* DNA polymerase. The PCR products were detected by gel electrophoresis in 0.8% agarose gels, stained using SuperRed/GelRed, and subsequently sequenced. Two primers were designed at the 5′ and 3′ ends of the *Il*-DyP4 gene with the following sequences: *Nco*I-DyPf and *Xho*I-DyPr, which contained the restriction sites *Nco*I and *Xho*I (underlined). The resulting PCR product was gel-purified, digested with *Nco*I and *Xho*I, and cloned into the corresponding site of the pET28a(+) vector to construct the recombinant plasmid pET28a-*Il*-DyP4. The plasmid was transformed into chemically competent *E. coli* Rosetta (DE3) cells and then confirmed by DNA sequencing. The gene clone and expression strain of *Il*-MnP6 were constructed using the same method (see Additional file 1: Table S1 for primers).

### *Il*-DyP4 and *Il*-MnP6 expression in *E. coli*

A positive transformant harboring pET28a-DyP4 was isolated as a single colony for gene expression. The transformant was cultured overnight at 37 °C in 5 mL LB medium containing 50 μg mL^−1^ kanamycin and 34 μg mL^−1^ chloramphenicol. The culture was then inoculated into fresh LB medium 400 mL containing kanamycin and chloramphenicol and grown at 37 °C and 200 rpm to an OD_600_ of approximately 0.6. IPTG was then added to a final concentration of 0.5 mM for induction for 4 h, bacterial pellets were obtained by centrifugation at 4 °C at 12,000×*g* for 10 min, and were resuspended in lysis buffer (0.02 mM PMSF, 50 mM Tris–HCl, and pH 7.5) in a quarter of the original culture volume. Bacteria were then lysed by sonication for 45 min. The lysed cells were centrifuged at 4 °C at 12,000×*g* for 30 min to obtain precipitation.

The *Il*-MnP6 protein was expressed according to the method of Chen et al. (2015).

### In vitro activation of *Il*-DyP4 and *Il*-MnP6

*Il*-DyP4 was expressed in the form of inclusion bodies and was solubilized in 10 mL 50 mM Tris–HCl (pH 8.0) containing 8 M urea, 1 mM EDTA, for 3 h at 4 °C to complete solubilization of the *Il*-DyP4 polypeptide. The refolding reaction system included 2 mM EDTA, 20 μM hemin, and 1 M urea, and had a pH of 6.0.

To investigate the optimal activation conditions for *Il*-DyP4 protein refolding, 0.2 mL protein (1.2 mg mL^−1^) was taken and optimized in 5-mL eppendorf tubes. Variable parameters include pH (1.0–8.0), EDTA (0–8 mM), hemin (0–60 μM), and urea (0.1–2.5 M). Refolding was performed at 4 °C for 24 h.

Larger-scale refolding assays were performed using the optimized conditions found in the small-scale experiments. The supernatant, including active *Il*-DyP4, was dialyzed against 10 mM sodium acetate (pH 6) for subsequent purification. The insoluble material was eliminated by centrifugation at 12,000×*g* for 30 min.

The refolding results were evaluated by measuring the *Il*-DyP4 residual enzyme activity using ABTS as reducing substrate according to the enzyme activity assay. Similarly, recombinant *Il*-MnP6 was expressed in the form of inclusion bodies. In vitro refolding was performed according to the method of Wang et al. (2016).

### *Il*-DyP4 and *Il*-MnP6 activity assays

*Il*-DyP4 activity was estimated spectrophotometrically by the oxidation of 1.25 mM ABTS to its cation radical (ε_418_ = 36,000 M^−1^ cm^−1^) in 100 mM sodium tartrate buffer at pH 3.5 at 25 °C.

*Il*-MnP6 activity was determined using two substrates. The first was determined based on the oxidation of Mn^2+^ to Mn^3+^ at 25 °C and 240 nm (ε_240_ = 6500 M^−1^ cm^−1^) at pH 4.5 in 0.11 M sodium lactate buffer (Salvachúa et al. 2013). The second was followed by the oxidation of 1.25 mM DMP to its cation radical (ε_469_ = 27,500 M^−1^ cm^−1^) in 100 mM sodium tartrate buffer containing 1 mM MnSO_4_, at pH 4 at 55 °C.

In all cases, peroxidase activity assays were carried out in the presence of 0.1 mM H_2_O_2_ and were carried out in triplicate. Control treatments without H_2_O_2_ and/or without enzyme were also performed. One unit (U) of peroxidase oxidized 1 μmol of substrate/min, and units were calculated based upon U mg^−1^ of protein per mL^−1^ of enzyme solution.

### Purification of DyP and MnP and mass characterization

Refolded solutions containing recombinant *Il*-DyP4 and *Il*-MnP6 were purified using Ni–NTA affinity chromatography. Sodium dodecyl sulfate polyacrylamide gel electrophoresis (SDS-PAGE) was performed using a 12% Tris–HCl separation gel, and samples were then stained with Coomassie Brilliant Blue R-250.

Furthermore, purified proteins were identified by mass spectrometry. First, purified recombinant *Il*-DyP4 and *Il*-MnP6 proteins were excised from the gel and digested with trypsin. Subsequently, the resulting peptide mixtures were analyzed by liquid chromatography–mass spectrometry (LC–MS) using a Proteome X-LTQ mass spectrometer (Thermo Fisher Scientific, Waltham, MA, USA). All obtained peptides were compared with the predicted amino acid sequence of *Il*-DyP4 and *Il*-MnP6.

### Effect of pH and temperature on DyP and MnP activity and stability

The optimum pH for substrate oxidation activity of *Il*-DyP4 and *Il*-MnP6 was estimated at 25 °C in 0.11 M citrate–phosphate buffer (pH 2.2–8.0) or Tris–HCl buffer (pH 8.6–9.0). To evaluate the pH stability, the enzymes were incubated at 4 °C for 12 h at different pH (2.2–9.0).

The optimum temperature for *Il*-DyP4 activity was measured from 0 to 60 °C in appropriate increments in 0.1 M sodium tartrate buffer (pH 3.5). The optimum temperature for *Il*-MnP6 activity was measured from 0 to 85 °C in appropriate increments in 0.11 M sodium lactate buffer (pH 4.5). To evaluate the thermal stability of *Il*-DyP4 and *Il*-MnP6, the enzymes were incubated at temperatures ranging from 4 to 65 °C in appropriate increments for 12 h.

The residual enzyme activities of *Il*-DyP4 and *Il*-MnP6 were determining using ABTS and Mn^2+^, respectively, as substrates.

### Substrate specificities

Eight different substrates [H_2_O_2_, ABTS (ε_418_ = 36,000 cm^−1^ M^−1^), Mn^2+^ (ε_240_ = 6500 cm^−1^ M^−1^), DMP (ε_469_ = 27,500 cm^−1^ M^−1^), guaiacol (ε_456_ = 12,100 cm^−1^ M^−1^), VA (ε_310_ = 9300 cm^−1^ M^−1^), RBlue19 (ε_595_ = 10,000 cm^−1^ M^−1^), RBlack 5 (ε_598_ = 30,000 cm^−1^ M^−1^)] were used to study the substrate specificities of *Il*-DyP4 and *Il*-MnP6 in 0.1 M sodium tartrate. The reactions were initiated by the addition of 0.1 mM H_2_O_2_. In the MnP reaction system, 1 mM Mn^2+^ was added. The oxidation activity for these substrates was measured based on the molar absorbance of the corresponding reaction product at the specific wavelength.

Steady-state kinetic constants of *Il*-DyP4 or *Il*-MnP6 were determined spectrophotometrically using a UV–visible spectrophotometer (Shanghai, China). First, the effect of pH and temperature on the activity for oxidation of eight different substrates by both enzymes were investigated, respectively, and then the optimal values were used to determine their kinetic constants (*K*_*m*_, *k*_*cat*_ and *k*_*cat*_/*K*_*m*_) by the hyperbolic, non-linear least squares method.

### H_2_O_2_ stability and the effects of compounds on enzyme activity

The H_2_O_2_ stability of both enzymes was determined by incubating 25 nM of enzyme for 30 min at 25 °C in the presence of increasing H_2_O_2_ enzyme concentrations (0–10 mM). The residual activities were then monitored using the method for assaying enzyme activity.

Additionally, under the specified enzyme activity measurement conditions, *Il*-DyP4 used ABTS as a substrate, whereas *Il*-MnP6 used DMP as a substrate. Residual enzyme activity was determined in the presence and absence of three different concentrations (0.1, 1, and 10 mM) of different cations (Co^2+^, Ba^2+^, Cd^2+^, Mn^2+^, Na^+^, Ca^2+^, Mg^2+^, Zn^2+^, Ni^2+^, Cu^2+^, Fe^2+^, Fe^2+^, and Al^3+^), thiourea, EDTA, DTT, and β-mercaptoethanol. Enzymes were incubated with these compounds for 5 min before the activity measurement.

### Spectroscopic characterization of *Il*-DyP4 and *Il*-MnP6

Far-UV CD spectra measurements were implemented with a MOS-500 CD spectrometer (Bio-Logic, Grenoble, France), with a 1-mm light path cell. The protein concentration was 0.1 mg mL^−1^ in 0.15 M phosphate buffer at pH 6.5. The CD spectra were recorded using a 2-mm bandwidth in the far-UV region (190–250 nm) at room temperature.

UV–Vis spectra were obtained in the 200–700 nm region at room temperature using a DU 730 UV spectrophotometer (Beckman Coulter, Brea, CA, USA); the *Il*-DyP4 concentration was 0.58 mg mL^−1^ in 0.1 M phosphate buffer at pH 6.0 and that of *Il*-MnP6 was 1.26 mg mL^−1^ in 0.1 M sodium acetate buffer at pH 5.9.

### *Il*-DyP4 and *Il*-MnP6 electrochemical experiments

Cyclic voltammetry (CV) was used to determine the redox potential of *Il*-DyP4 and *Il*-MnP6. Pyrolytic graphite electrode (PGE), platinum wire, and Ag/AgCl were used as the working electrode, the counter electrode, and reference electrode, respectively (Mendes et al. 2015). The first step the PGE surface was polished with Al_2_O_3_ on sandpaper (2000 mesh); secondly, the electrode was thoroughly ultrasonicated with water and ethanol for 5 min each; thirdly, 5 μg protein was dropped onto the PGE, and maintained at 4 °C for 20 h to allow the protein to fully adsorb to the electrode surface. Finally, the electrode was cleaned with distilled water, measured for a scan rate at 50 mV s^−1^, and the potential was then cycled between − 1.0 and + 0.6 V.

### Dye decolorization

The decolorization ability of *Il*-DyP4 or *Il*-MnP6 towards five different classes of dye (anthraquinone dyes, azo dyes, phenazine dyes, triphenylmethane dyes, and aniline dyes) was measured using a spectrophotometer. Reaction mixtures contained 100 mM sodium tartrate buffer (pH 3.5, 4.0 or 4.5), 25 nM of purified enzyme and dyes (at a concentration of 25–200 μM); the concentration ratio of H_2_O_2_/dye was 4:1. Besides, 1 mM Mn^2+^ was supplemented in the *Il*-MnP6 reaction system. Reactions were subsequently performed for 30 min at 35 °C, and were then examined for the level of decolorization by the two enzymes.

Decolorization assays of polymeric dye Poly R-478 were performed to reveal the ability of *Il*-DyP4 or *Il*-MnP6 to degrade lignin. The reaction mixture contained sodium tartrate buffer (100 mM), Poly R-478 dye (0.01%), and *Il*-DyP4 (100 nM) or *Il*-MnP6 (100 nM) in a total volume of 1 mL (MnP reaction system added 1 mM Mn^2+^). The reaction was initiated by the addition of H_2_O_2_ (0.2 mM) and the mixture was incubated at different pH conditions at 35 °C for 30 min. Dye decolorization was measured using a spectrophotometer at 520 nm, which is the maximum visible absorbance of Poly R-478. Control samples, without enzyme or H_2_O_2_, were done in parallel under identical conditions.

The decolorization percentage was calculated according to Eq. 1.

1 $${\text{Decolorization}}\left( \% \right)\, = \,\frac{{A_{0} - A_{t} }}{{A_{0} }}\, \times \,100\%$$

where *A*_0_ is the initial absorbance at λ_max_ (nm) and *A*_t_ refers to the absorbance at λ_max_ (nm) at reaction time t. The data were the mean values of triplicate experiments.

## Results

### DyP and MnP cDNAs analyses

Amino acid sequence alignments of five DyPs (*Il*-DyP1–5: accession numbers MH120197, MH120198, MH120199, MG209114, and MH120196, respectively) from *I. lacteus* F17 displayed typical characteristic GXXDG conserved motifs (Additional file 1: Fig. S1). Some crucial role of the conserved amino acid residues were shown in colors, such as the proximal histidine residue as well as the distant aspartic acid and arginine residues. There was a 57–83% shared sequence identity among the five DyPs (Additional file 1: Table S2). Moreover, alignment analysis of conserved amino acid sites in 13 MnPs (*Il*-MnP1–13: KC811382, MH120200, MH120201, MH120202, MH120203, MG209112, MH120204, MH120205, MH120206, MH120207, MH120208, MH120209, and MH120210, respectively) from *I. lacteus* F17 was also performed (Additional file 1: Fig. S2). Likewise, some crucial role of the conserved amino acid residues was shown in colors, including Mn^2+^-binding and Ca^2+^-binding amino acid ligands etc. There was a 49–99% shared sequence identity among the 13 MnPs (Additional file 1: Table S3).

To understand the essential characteristics and biochemical properties of these two different types of enzyme, we tested DyPs and MnPs expression levels in *I. lacteus* F17 by culturing this organism in CPDA liquid shake cultures (28 °C). Genes encoding *Il*-DyP4 and *Il*-MnP6 were found to be the predominant expressed genes. Therefore, *Il*-DyP4 (MG209114) and *Il*-MnP6 (MG209112) were selected for further research.

According to the ExPASy (Gasteiger et al. 2003) server prediction online, *Il*-DyP4 contained 502 amino acids, with a molecular weight of 54.5 kDa and a theoretical pI of 5.06. The signal peptide of *Il*-DyP4 was confirmed to contain 52 amino acids (Salvachúa et al. 2013). In addition, *Il*-DyP4 showed 58% identity with *Bad*DyP (GenBank: BAA77283.1) from *Bjerkandera adusta*, 42% identity with *Pleurotus ostreatus* DyP (GenBank: CAK55151.1), 40% identity with *T. versicolor* DyP (GenBank: EIW57847.1), and < 33% identity with DyPs from bacteria, such as *E. coli* (PDB 5GT2_A), *Shewanella oneidensis* (PDB 2HAG_A), and *Bacteroides thetaiotaomicron* (PDB 2GVK_A). According to the ExPASy server, *Il*-MnP6 contained 359 amino acids, with a molecular weight of 38.2 kDa and a theoretical pI of 4.94. Using SignalP 4.1, the signal peptide of *Il*-MnP6 was predicted to be 23 amino acids long. In addition, *Il*-MnP6 showed 73% identity with MnP3 (GenBank: CAD92855.1) from *Phlebia radiata*, 72% identity with MnP (GenBank: CAG33918.4) from *T. versicolor*, and 55% identity with MnP (GenBank: AAB30859.1) from *P. chrysosporium*. Moreover, *Il*-MnP6 contained eight cysteines, and formed four disulfide bonds, and its amino acid sequence revealed an 89% identity with previously reported *Il*-MnP1 (GenBank:AGO86670.2) from *I. lacteus* F17, indicating that it is a short-type MnP (Chen et al. 2015). As such, the other 11 MnPs in *I. lacteus* F17 genome also belonged to the short MnP subfamily, based on the analyses of their amino acid sequences.

To analyze the structural properties of the two enzymes, molecular models of the deduced mature proteins were built (Additional file 1: Fig. S3). For *Il*-DyP4, the crystal structure of *B. adusta* DyP (PDB 3afv) (sequence identity 62.19%) was used as a template. For *Il*-MnP6, the crystal structure of *Pleurotus eryngii* VP (PDB 2boq) was used as a template (sequence identity 70.61%). In addition, the presence of the amino acids residues mentioned above was confirmed in the two structural models (Additional file 1: Fig. S3).

### Expression and in vitro activation of *Il*-DyP4 and *Il*-MnP6

The cDNA sequences encoding *Il*-DyP4 and *Il*-MnP6 were amplified by PCR and inserted into the expression vector pET28a(+). The resulting recombinant constructs were then transformed into *E. coli* Rosetta (DE3) cells for heterologous expression.

**Fig. 1:**
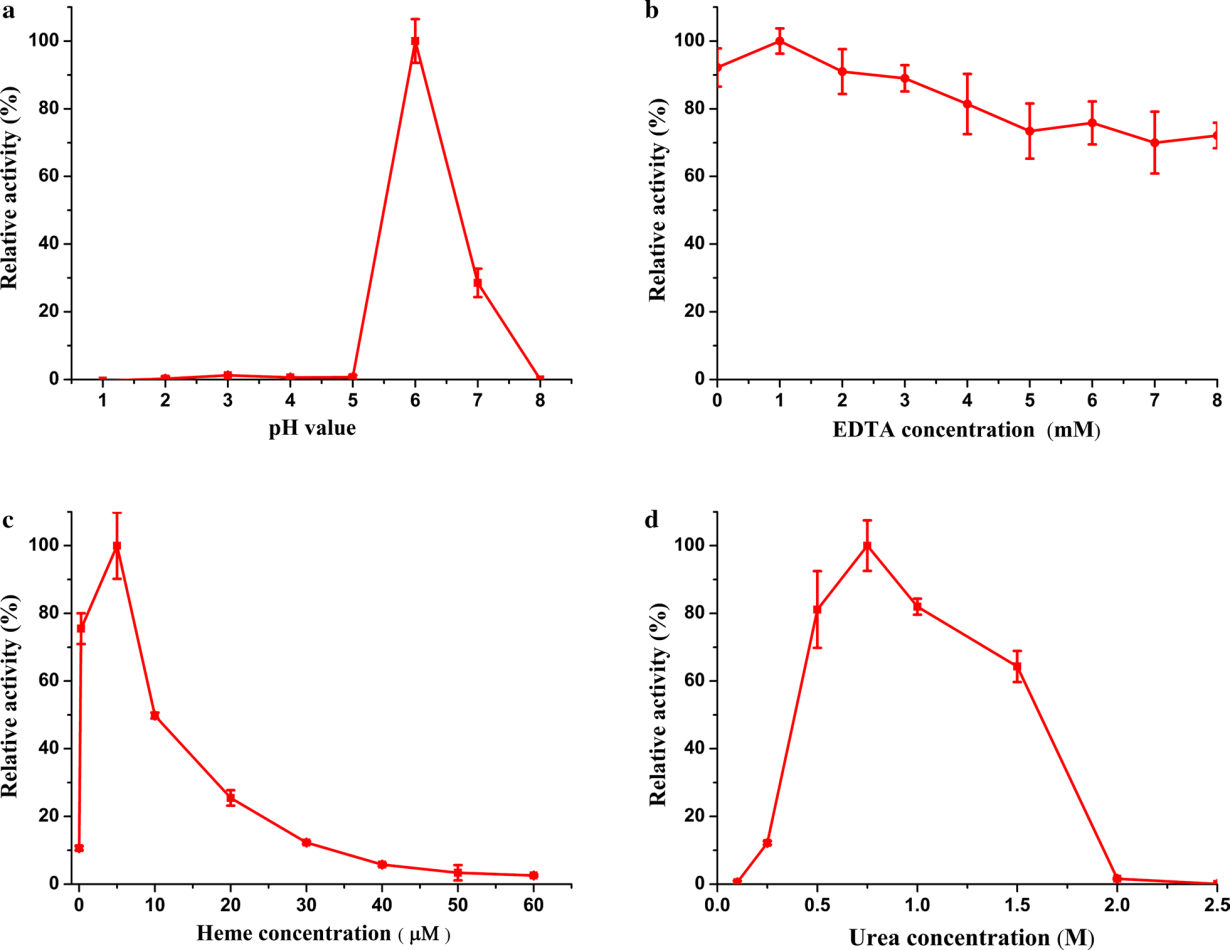
The effect of several parameters on the refolding of recombinant *Il*-DyP4. **a** pH, **b** EDTA concentration, **c** hemin concentration, and **d** urea concentration were systematically examined. All reactions were performed with 1.2 mg mL^−1^ protein in 50 mM phosphate buffer. The reactions took place in the dark for 24 h at 4 °C. The residual activity of *Il*-DyP4 was measured according to enzyme activity assay. Deviation values are standard deviations based on triplicate determinations

The optimum enzyme activity achieved for *Il*-DyP4 was 10.8 U mg^−1^, using the following optimal refolding conditions: 1 mM EDTA, 5 μM hemin, 0.75 M urea in 50 mM phosphate (pH 6) (Fig. 1a–d).

The optimum enzyme activity of *Il*-MnP6 was 2 U mg^−1^, using the following optimal refolding conditions: 1.5 M urea, 150 mM CaCl_2_, 25 μM hemin, 10% glycerol, 0.5 mM GSSG, 0.05 mM MnSO_4_, 20 mM KCl in 50 mM Tris–HCl (pH 8.5).

### Purification of *Il*-DyP4 and *Il*-MnP6 and mass characterization

Two recombinant proteins with six His-tags at the C terminus were purified using Ni–NTA affinity chromatography, and exhibited a single band on SDS-PAGE (Additional file 1: Fig. S4). Purified *Il*-DyP4 had a molecular weight of 54.0 kDa, close to the 54.5 kDa predicted. The final recovery, which was obtained from the total activity, was approximately 85% (Table 1). The specific activity of purified *Il*-DyP4 was 140 U mg^−1^. Purified *Il*-MnP6 had a molecular weight of 44.6 kDa, close to the 38.2 kDa predicted (Additional file 1: Fig. S4). The final recovery of *Il*-MnP6 was almost 60% (Table 1). The specific activity of purified *Il*-MnP6 was 23.8 U mg^−1^.

**Table 1:** Isolation and purification of *Il*-DyP4 and *Il*-MnP6 from inclusion bodies

	Sample	Protein concentration (mg mL^−1^)	Protein (mg)	Specific activity (U mg^−1^)	Total activity (U)	Yield (%)
DyP	Inclusion body	8.78	87.8			
	Dialyzed	0.24	28.2	10.8	305	100
	Ni–NTA	0.0093	1.87	140	261	86
MnP	Inclusion body	4.45	22.2			
	Dialyzed	0.23	8.1	2	16.2	100
	Ni–NTA	0.0082	0.41	23.8	9.8	60

The purified *Il*-DyP4 was analyzed by mass spectrometry. These peptides accurately matched the deduced amino acid sequence of *Il*-DyP4 (see Additional file 1: Table S4). The first fragment (MGSAGNDSLPFENIQGDILVGMKK) was the N-terminus amino acid sequence, which has 100% identity with the native *I. lacteus* DyP (Salvachúa et al. 2013).

Similarly, the purified *Il*-MnP6 was also analyzed by mass spectrometry. These peptides accurately matched the deduced amino acid sequence of *Il*-MnP6 (see Additional file 1: Table S5). Thus, these data confirmed the successful expression of *Il*-DyP4 and *Il*-MnP6 in *E. coli* Rosetta (DE3).

### Effects of pH and temperature on the activity and stability of *Il*-DyP4 and *Il*-MnP6

**Fig. 2:**
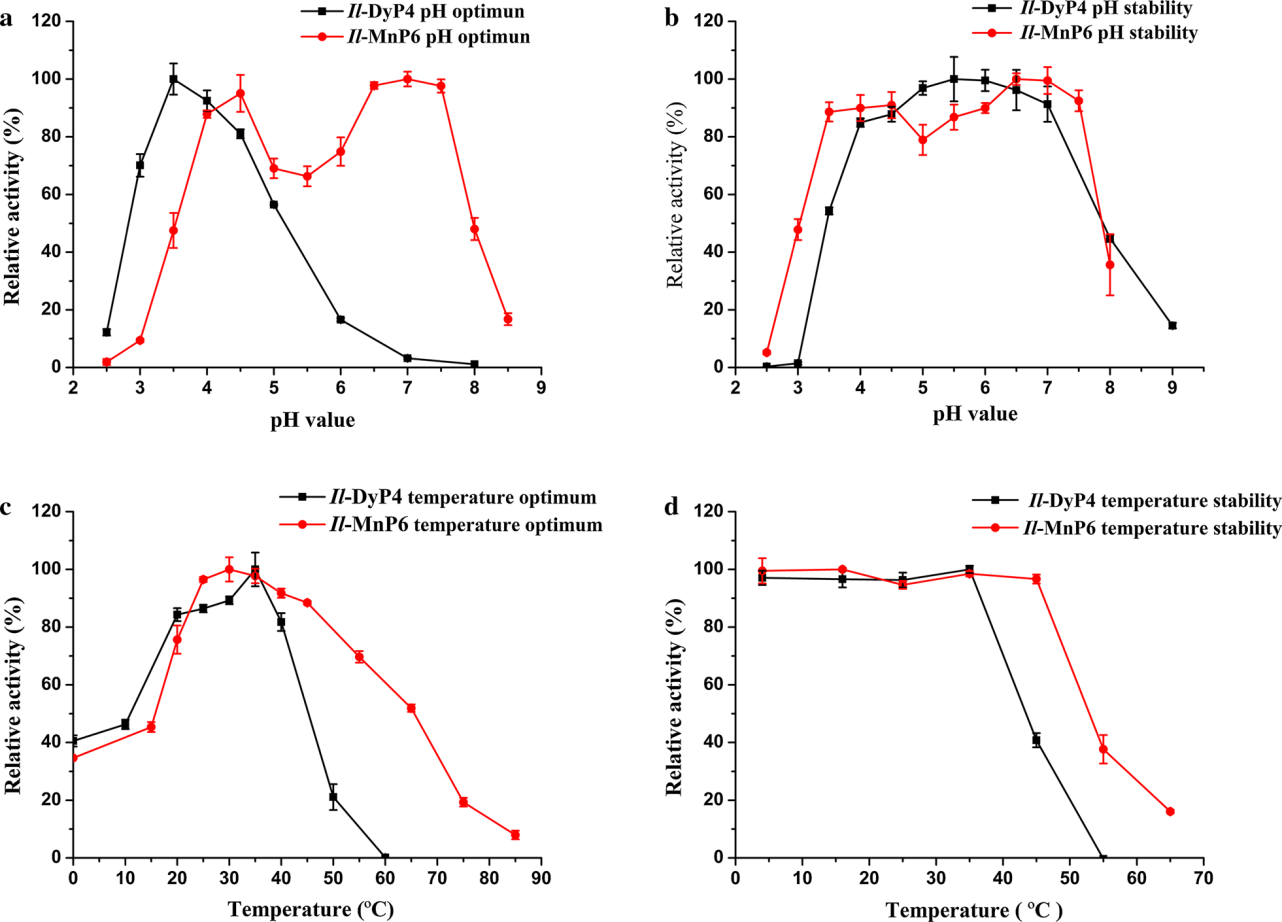
Effects of pH and temperature on the activity and stability of *Il*-DyP4 and *Il*-MnP6. And *Il*-DyP4 and *Il*-MnP6 activities at the beginning were considered to be 100%. Deviation values are standard deviations based on triplicate determinations. **a** The pH optimum of *Il*-DyP4 and *Il*-MnP6. The *Il*-DyP4 and *Il*-MnP6 activities were determined in the citrate–phosphate buffer, pH (2.2–8.0) and Tris–HCl buffer (pH 8.5) at 25 °C, respectively. **b** The pH stability of *Il*-DyP4 and *Il*-MnP6. *Il*-DyP4 and *Il*-MnP6 was incubated for 12 h at 25 °C in various pH values in citrate–phosphate (2.2–8.0) or Tris–HCl buffer (9.0). The residual activity of *Il*-DyP4 and *Il*-MnP6 was measured according to their enzyme activity assay. **c** The temperature optimum of *Il*-DyP4 and *Il*-MnP6. The enzyme reaction of *Il*-DyP4 was performed in 0.1 M sodium tartrate buffer, pH 3.5 at 0–60 °C. The enzyme reaction of *Il*-MnP6 was performed in 0.11 M sodium lactate buffer, pH 4.5 at 0–85 °C. **d** The temperature stability of *Il*-DyP4 and *Il*-MnP6. *Il*-DyP4 and *Il*-MnP6 were incubated for 12 h at 4–65 °C. The residual activity of *Il*-DyP4 and *Il*-MnP6 was measured according to their enzyme activity assay

The optimal pH of *Il*-DyP4 for ABTS oxidation was 3.5 (Fig. 2a). During incubation of *Il*-DyP4 in different pH buffers for 12 h, the enzyme retained more than 80% of its enzymatic activity at pH 4–7 and was most stable at pH 5.5 (Fig. 2b). Unlike *Il*-DyP4, the optimum pH of *Il*-MnP6 showed double peaks (4.5 and 7.0), and used Mn^2+^ as a substrate. *Il*-MnP6 also retained > 75% of its enzymatic activity at a wider range of pH (3.5–7.5), indicating that the pH stability of *Il*-MnP6 was superior to that of *Il*-DyP4.

*Il*-DyP4 exhibited the highest enzymatic activity at 35 °C and retained 90% of its enzymatic activity after incubation at temperatures below 35 °C for 12 h (Fig. 2c, d). *Il*-MnP6 showed maximal enzymatic activity at 30 °C and retained > 90% of its enzymatic activity below 45 °C for 12 h.

### Substrate specificities of *Il*-DyP4 and *Il*-MnP6

Significant differences were recorded in the substrate specificities of *Il*-DyP4 and *Il*-MnP6 on eight representative substrates (Table 2). For heme-containing peroxidases, H_2_O_2_ was used as an electron-accepting substrate to oxidize a variety of compounds. The results showed that the catalytic efficiency of *Il*-DyP4 in the reduction of H_2_O_2_ was an order of magnitude higher than that of *Il*-MnP6. For ABTS oxidation, the catalytic efficiency (*k*_*cat*_/*K*_*m*_) of *Il*-DyP4 was up to 1.3 × 10^8^ s^−1^ M^−1^, two orders of magnitude higher than that of *Il*-MnP6. For Mn^2+^ oxidation, which is often used as a substrate for MnP, the catalytic efficiency (*k*_*cat*_/*K*_*m*_) of *Il*-DyP4 was 3.0 × 10^5^ s^−1^ M^−1^, which was an order of magnitude lower than that of *Il*-MnP6. Phenolic substrates, such as DMP and guaiacol, which were commonly used as universal substrates for heme peroxidase, displayed similar catalytic efficiency for *Il*-DyP4 and *Il*-MnP6. VA, a nonphenolic compound, was also assayed, revealing the catalytic efficiency of *Il*-DyP4 to be 5.2 × 10^3^ s^−1^ M^−1^, although no activity was detected for *Il*-MnP6 in 0.1 M sodium tartrate, pH 3.5. For two dyes tested, the catalytic efficiency of *Il*-DyP4 oxidizing anthraquinone dye RBlue 19 was 4.0 × 10^7^ s^−1^ M^−1^, which was three orders of magnitude higher than that of *Il*-MnP6. The catalytic efficiency of *Il*-DyP4 oxidizing azo dyes RBlack 5 was 1.7 × 10^6^ s^−1^ M^−1^, which was two orders of magnitude higher that of *Il*-MnP6.

**Table 2:** Kinetic parameters—*K*_*m*_ (μM), *k*_*cat*_ (s^−1^), and *k*_*cat*_/*K*_*m*_ (s^−1^M^−1^) of *Il*-DyP4 and *Il*-MnP6 for substrates oxidation obtained based on the optimal pH and temperature (°C)

	*Il*-DyP4				*Il*-MnP6			
	pH	Temperature	Kinetic constants		pH	Temperature	Kinetic constants	
H_2_O_2_^a^	3.5	35	*K*_*m*_	163 ± 20.8	4.5	30	*K*_*m*_	194 ± 18
			*k*_*cat*_	(1.2 ± 0.08) × 10^4^			*k*_*cat*_	879 ± 48
			*k*_*cat*_/*K*_*m*_	(7.6 ± 0.5) × 10^7^			*k*_*cat*_/*K*_*m*_	(4.5 ± 0.3) × 10^6^
ABTS	3.5	35	*K*_*m*_	62 ± 11	3.5	45	*K*_*m*_	83 ± 6.5
			*k*_*cat*_	8356 ± 747			*k*_*cat*_	245 ± 7
			*k*_*cat*_/*K*_*m*_	(1.3 ± 0.1) × 10^8^			*k*_*cat*_/*K*_*m*_	(3.0 ± 0.09) × 10^6^
Mn^2+^	4.5	35	*K*_*m*_	2687 ± 463	4.5	30	*K*_*m*_	129 ± 8
			*k*_*cat*_	806 ± 94			*k*_*cat*_	369 ± 6.1
			*k*_*cat*_/*K*_*m*_	(3.0 ± 0.4) × 10^5^			*k*_*cat*_/*K*_*m*_	(2.9 ± 0.3) × 10^6^
DMP	4	35	*K*_*m*_	58 ± 3	4.5	55	*K*_*m*_	53 ± 8.6
			*k*_*cat*_	4896 ± 131			*k*_*cat*_	989 ± 100
			*k*_*cat*_/*K*_*m*_	(8.4 ± 0.2) × 10^7^			*k*_*cat*_/*K*_*m*_	(1.9 ± 0.2) × 10^7^
Guaiacol	4	45	*K*_*m*_	24 ± 7.5	4	45	*K*_*m*_	2.6 ± 0.6
			*k*_*cat*_	413 ± 34			*k*_*cat*_	184 ± 7
			*k*_*cat*_/*K*_*m*_	(1.7 ± 0.1) × 10^7^			*k*_*cat*_/*K*_*m*_	(7.1 ± 0.3) × 10^7^
VA	3.5	45	*K*_*m*_	(2.1 ± 0.98) × 10^4^	–^b^	–	*K*_*m*_	–^c^
			*k*_*cat*_	108 ± 47			*k*_*cat*_	–
			*k*_*cat*_/*K*_*m*_	(5.2 ± 2.3) × 10^3^			*k*_*cat*_/*K*_*m*_	–
RBlue 19	4.5	35	*K*_*m*_	133 ± 34	3	35	*K*_*m*_	81 ± 39.5
			*k*_*cat*_	5345 ± 921			*k*_*cat*_	7.3 ± 2.6
			*k*_*cat*_/*K*_*m*_	(4.0 ± 0.7) × 10^7^			*k*_*cat*_/*K*_*m*_	(9.0 ± 3.2) × 10^4^
RBlack 5	4	35	*K*_*m*_	159 ± 61	3	35	*K*_*m*_	4.4 ± 0.7
			*k*_*cat*_	267 ± 96			*k*_*cat*_	0.06 ± 0.003
			*k*_*cat*_/*K*_*m*_	(1.7 ± 0.6) × 10^6^			*k*_*cat*_/*K*_*m*_	(1.5 ± 0.07) × 10^4^

Means and 95% confidence limits

^a^Activities were measured with 1.25 mM ABTS and 1 mM Mn^2+^ by *Il*-DyP4 and *Il*-MnP6, respectively

^b^Not detected

^c^Experimental condition: [VA] = 1 mM, [MnP] = 25 nM, [H_2_O_2_] = 0.1 mM, [MnSO_4_] = 1.0 mM, [Tartrate buffer] = 100 mM, pH = 3.5, temperature = 45 °C, reaction time = 60 min

### Effects of H_2_O_2_ and chemicals on *Il*-DyP4 and *Il*-MnP6 activity

In general, fungal heme-containing peroxidases depend on H_2_O_2_ to start the catalytic cycle of the reaction, although the enzymes are prone to inactivation in the presence of excess H_2_O_2_. The H_2_O_2_ stability of *Il*-DyP4 and *Il*-MnP6 showed that the enzyme activity of *Il*-DyP4 was reduced by 60% as the H_2_O_2_ concentration increased from 0.1 to 4 mM, whereas the enzyme activity of *Il*-MnP6 was reduced by 10%. However, when the H_2_O_2_ concentration was increased from 4 to 10 mM, the enzyme activity of *Il*-DyP4 was reduced by only 10%, whereas 80% of the enzymatic activity of *Il*-MnP6 was lost rapidly. Therefore, *Il*-DyP4 was less resistant to low H_2_O_2_ concentrations, and more resistant to high H_2_O_2_ concentrations, and vice versa for *Il*-MnP6.

The effects of three concentrations of several metal ions and other compounds on *Il*-DyP4 and *Il*-MnP6 were also assayed (Table 3). For Mn^2+^, Na^+^, Mg^2+^, Zn^2+^, Ni^2+^, Cu^2+^, and Al^3+^, no loss of enzyme activity was observed for *Il*-DyP4 and *Il*-MnP6 within the tested concentrations. When the concentrations of Co^2+^ and Ca^2+^ were increased to 10 mM, *Il*-DyP4 was significantly inhibited, whereas no significant effect was observed on *Il*-MnP6. Both Fe^3+^ and Fe^2+^ significantly inhibited the activity of both enzymes, except at low concentrations of Fe^3+^ (0.1 mM).

**Table 3:** Effects of different chemicals on the activity of *Il*-DyP4 and *Il*-MnP6

Chemicals	*Il*-DyP4^a^			*Il*-MnP6^b^		
	Relative enzyme activity (%)			Relative enzyme activity (%)		
	0.1 mM	1 mM	10 mM	0.1 mM	1 mM	10 mM
Co^2+^	97	76	21	107	107	111
Ba^2+^	101	103	88	106	95	115
Cd^2+^	100	103	87	103	98	51
Mn^2+^	101	100	102	67	100	106
Na^+^	99	93	96	103	96	98
Ca^2+^	87	79	47	94	97	90
Mg^2+^	102	100	102	93	105	108
Zn^2+^	88	86	72	91	93	85
Ni^2+^	100	106	102	98	97	92
Cu^2+^	104	109	98	96	98	50
Fe^2+^	8	9	1	19	0	0
Fe^3+^	104	13	2	100	37	0
Al^3+^	107	107	69	100	91	22
Thiourea	99	93	33	95	82	19
EDTA	99	107	87	93	8	4
DTT	99	1	0	25	0	0
β-Mercaptoethanol	92	45	0	15	0	0

^a^ABTS oxidation by the *Il*-DyP4

^b^DMP oxidation by the *Il*-MnP6

DTT, thiourea, and β-mercaptoethanol had little effect on *Il*-DyP4 activity at lower concentrations (0.1 mM), whereas *Il*-MnP6 activity was completely or partially inhibited when using 0.1 mM and 1 mM of these compounds. There was no change in *Il*-DyP4 activity at the three EDTA concentrations tested, whereas *Il*-MnP6 activity declined rapidly.

### Spectroscopic characterization of *Il*-DyP4 and *Il*-MnP6

Circular dichroism (CD) spectra measurement in the far-UV region is often used to observe the secondary structures of proteins. The far-UV CD spectra of *Il*-DyP4 and *Il*-MnP6 exhibited two negative ellipticity bands at 208 and 222 nm, respectively (Additional file 1: Fig. S5), indicating α-helical structure characteristics, but with significant differences in content. The α-helix and β-sheet contents of *Il*-DyP4 and *Il*-MnP6 were calculated using the K2D program. The results showed that the α-helical content of *Il*-DyP4 (≥ 9%) was lower than that of *Il*-MnP6 (≥ 17%), whereas the β-sheet content of *Il*-DyP4 (≥ 43%) was higher than that of *Il*-MnP6 (≥ 31%).

**Fig. 3:**
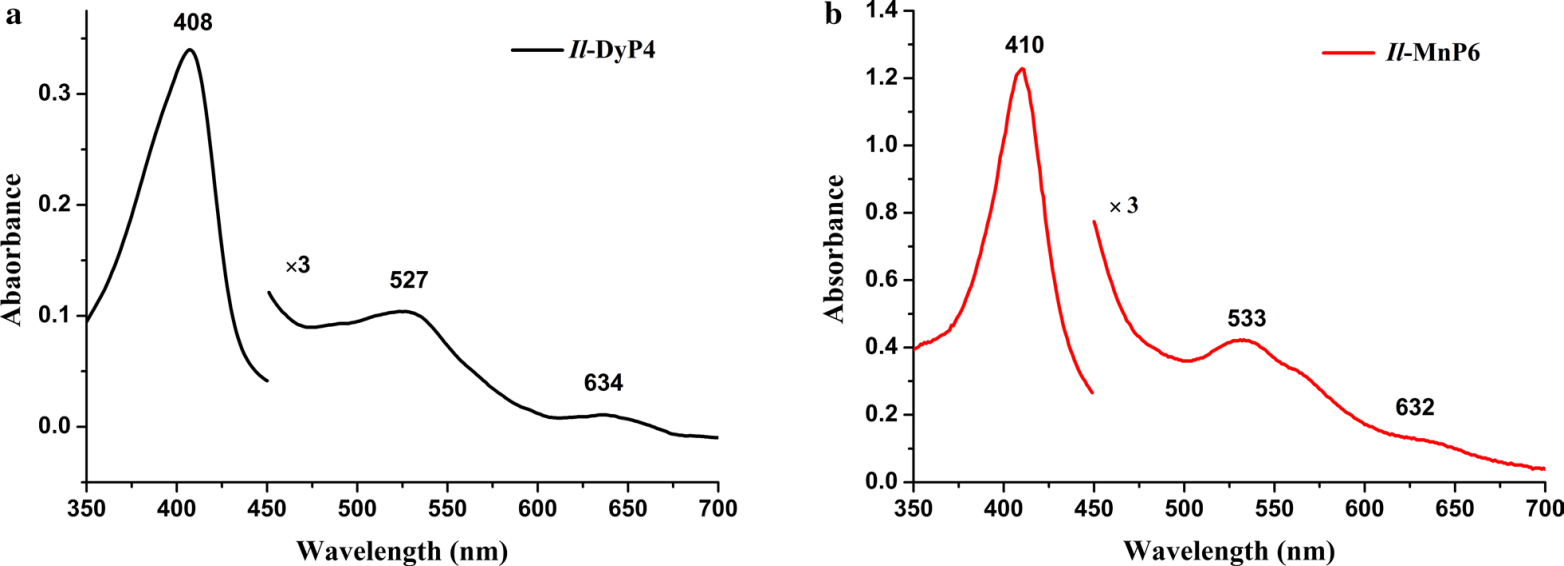
**a** The UV–Vis absorbance spectra of *Il*-DyP4. **b** The UV–Vis absorbance spectra of *Il*-MnP6. The region between 450 and 700 nm has been expanded (×3 absorbance)

The UV–Vis spectrum of *Il*-DyP4 (Fig. 3a) was similar to that of *Il*-MnP6 (Fig. 3b). *Il*-DyP4 exhibited a Soret peak at 408 nm, a Q band at 527 nm, and a charge transfer (CT) band at 634 nm, while *Il*-MnP6 showed a Soret peak at 410 nm, a Q band at 533 nm, with a narrow CT band at 632 nm. The Reinheitszahl (R_Z_) values (calculated from the A_408_/A_280_ and A_410_/A_280_ absorption ratios) for *Il*-DyP4 and *Il*-MnP6 were 1.5 and 3.17, respectively.

### Redox properties of *Il*-DyP4 and *Il*-MnP6

The midpoint redox potential of the Fe^3+^/Fe^2+^ couple ($E^{m^{\prime}}_{\text{Fe}^{3+}/{\text{Fe}^{2+}}}$) at different pH values (3.5, 6.5, and 8) were estimated by CV for *Il*-DyP4 and *Il*-MnP6 (Table 4). *Il*-DyP4 obtained the highest midpoint redox potential ($E^{m^{\prime}}_{\text{Fe}^{3+}/{\text{Fe}^{2+}}}$= 27 ± 10 mV) at pH 3.5, decreasing to a lower potential ($E^{m^{\prime}}_{\text{Fe}^{3+}/{\text{Fe}^{2+}}}$= − 232 ± 10 mV) at pH 8.0. Similarly, *Il*-MnP6 obtained the highest midpoint redox potential ($E^{m^{\prime}}_{\text{Fe}^{3+}/{\text{Fe}^{2+}}}$= − 75 ± 10 mV) at pH 3.5, decreasing to a lower potential ($E^{m^{\prime}}_{\text{Fe}^{3+}/{\text{Fe}^{2+}}}$= − 168 ± 10 mV) at pH 8.0. When the pH increased from 3.5 to 8.0, the midpoint redox potential of *Il*-DyP4 was decreased by 259 mV, whereas that of *Il*-MnP6 decreased by 93 mV.

**Table 4:** Midpoint redox potentials of *Il*-DyP4 and *Il*-MnP6 (vs. Ag/AgCl)

	$$E^{m^{\prime}}_{\text{Fe}^{3+}/{\text{Fe}^{2+}}}$$ (mV)		
pH	3.5	6.5	8.0
*Il*-DyP4	27 ± 10	− 87.5 ± 10	− 232 ± 10
*Il*-MnP6	− 75 ± 10	− 100 ± 10	− 168 ± 10

### Dye decolorization

Dyes are colorful organic compounds that are widely used in the food, textile, drug, and paper industries, often resulting in water pollution. The first DyP discovered was named because of its ability to decolorize various dyes (Kim and Shoda 1999). Accordingly, we selected 16 different dyes to examine the decolorization capability of *Il*-DyP4 and *Il*-MnP6 under pH 3.5, 4.0, and 4.5. The results revealed that the ability of *Il*-DyP4 to oxidize most of the anthraquinone dyes, azo dyes, phenazine dyes, triphenylmethane dyes, and aniline dyes was stronger than that of *Il*-MnP6 (Table 5), and also greater than that of *Tfu*DyP from *Thermobifida fusca* (van Bloois et al. 2010). In addition, the results revealed that *Il*-DyP4 was able to decolorize anthraquinone dyes more quickly than was *Il*-MnP6. Interestingly, direct yellow 8 did not show any significant decolorization in our experiments. Further decolorization experiments using this dye are in progress.

**Table 5:** Decolorization (%) of different kinds of synthetic dyes by *Il*-DyP4 and *Il*-MnP6

Nr.	Dye	λ_max_ (nm)	Concentration (μM)	*Il*-DyP4^a^			*Il*-MnP6^a^		
				pH 3.5	pH 4	pH 4.5	pH 3.5	pH 4	pH 4.5
*Anthraquinone dyes*									
1	Reactive blue 4	598	200	79.28	81.92	82.01	1.87	7.24	0.96
2	Reactive blue 5	600	100	74.06	82.73	83.61	12.40	18.07	8.26
3	Reactive blue 19	595	100	78.39	74.97	70.01	2.78	5.71	5.90
*Azo dyes*									
4	Direct sky blue 5B	598	100	80.62	81.45	77.91	16.95	59.72	63.75
5	Reactive black 5	598	50	60.57	59.30	40.86	0.22	0	0.69
6	Acid red 18	506	100	43.07	43.73	38.04	6.09	7.14	3.53
7	Reactive violet 5	570	100	92.16	90.81	80.96	19.97	21.32	1.10
8	Methyl orange	464	50	33.12	30.21	29.60	11.21	9.32	1.95
9	Direct yellow 8	392	25	2.62	4.66	2.04	0	0	0
10	Orange G	478	50	18.38	21.28	25.39	33.80	34.72	22.90
11	Orange yellow II	484	50	28.65	27.94	36.59	7.58	4.68	2.18
12	Orange yellow IV	445	50	62.55	60.07	60.16	13.23	10.40	4.26
13	Congo red	498	10	0	38.69	52.77	0	0	0
*Phenazine dyes*									
14	Neutral red	550	100	22.50	24.15	31.09	0	2.40	5.68
*Triphenylmethane dyes*									
15	Malachite green	618	50	84.80	82.43	72.53	24.74	28.03	24.69
*Aniline dyes*									
16	Basic fuchsin	542	25	45.10	39.75	23.86	3.46	3.63	0.25

^a^Percentage of dye decolorization after 30 min is based on the observed decrease in absorbance at λ_max_

In addition, we selected Poly R-478 to determine the ability of *Il*-DyP4 and *Il*-MnP6 to degrade lignin. The maximum efficiency of *Il*-DyP4 to decolorize Poly R-478 at an enzyme concentration of 100 nM was 15.76%, which was higher than the 0.66% of *Il*-MnP6 under pH 4.0 at 35 °C (Additional file 1: Fig. S6), indicating the *Il*-DyP4 had a better ligninolytic activity compared with *Il*-MnP6.

When the concentration of *Il*-DyP4 increased from 100 to 1000 nM, and the H_2_O_2_ concentration increased from 0.2 to 0.4 mM, the efficiency of *Il*-DyP4 to oxidize 0.01% Poly R-478 at pH 4 increased to 24%. When the concentration of *Il*-DyP4 was 1000 nM, and H_2_O_2_ concentration increased from 0.4 to 1.2 mM, the efficiency of *Il*-DyP4 to oxidize 0.01% Poly R-478 at pH 4 increased to 45%. The maximum decolorization percentage was 50% for *Il*-DyP4 for 1 h of reaction, and no increase in decolorization efficiency was observed 3 h after the initiation of the reaction.

## Discussion

*I. lacteus* is a cosmopolitan white-rot species that has become a model ligninolytic basidiomycete. The fungus can produce LiP, MnP, and laccase simultaneously, which is similar to *P. radiata* and *T. versicolor* (Novotný et al. 2009).

Recently, we published the analysis of the genome sequence of *I. lacteus* F17 (Yao et al. 2017) and updated five cDNAs encoding DyPs and 13 cDNAs encoding MnPs to GeneBank. Herein, we used the available information to further analyze the amino acid sequence consensus and important residues in these DyPs and MnPs, which belong to two different heme peroxidase families. Furthermore, among these enzymes, *Il*-DyP4 and the *Il*-MnP6 were selected and characterized to further investigate the basis of the ligninolytic system of *I. lacteus* F17.

Both recombinant enzymes were expressed as inclusion bodies in *E. coli* Rosetta (DE3) and active enzymes were achieved via in vitro refolding. For optimizing of *Il*-DyP4, the refolding solution needed the supplement of optimal concentrations of EDTA, hemin, and urea. Compared with *Il*-DyP4, the in vitro refolding process of *Il*-MnP6 was complicated and active *Il*-MnP6 was obtained by the refolding solution containing various factors, including urea, hemin, GSSG, CaCl_2_, glycerin, MnSO_4_, KCl, hemin, and Ca^2+^ at pH 8.0. Among these, a key factor of Ca^2+^ affected the reconstitution of this fungal MnP. These refolding differences could be ascribed to the different amino acid compositions and structures of the two proteins.

SDS-PAGE analyses indicated that purified *Il*-DyP4 was monomeric, and its molecular weight was consistent with that of reported DyPs (50–60 kDa) (Colpa et al. 2014). In addition, comparison of the N-terminal sequence of *Il*-DyP4 with that of native DyP from *I. lacteus* revealed their similarity. Likewise, *Il*-MnP6 purified by Ni–NTA affinity chromatography was also a monomer, and its molecular weight was almost identical to that of purified native MnP from *I. lacteus* F17 (Zhao et al. 2015).

In this study, the biochemical properties of the two heme peroxidases were investigated. Under acidic conditions at pH 3.5, *Il*-DyP4 was more robust for ABTS oxidation, which fell within the optimal pH range (pH 3–4.5) of most of the DyPs reported from fungi (Linde et al. 2014; Fernández-Fueyo et al. 2015; Behrens et al. 2016), and was similar to the optimal pH of native DyP from *I. lacteus* (Salvachúa et al. 2013). Intriguingly, compared with the acidic pH of fungi DyPs, the optimal pH of bacteria DyPs was 5.0–7.0 (Yu et al. 2014; Ramzi et al. 2015; Rahmanpour et al. 2016). The most distinguishing feature of DyP peroxidases is their powerful catalytic properties (Colpa et al. 2014). In the current study, *Il*-DyP4 exhibited a broad substrate range and a strong ability to oxidize different structures of compounds.

Although all the substrates tested except VA were oxidized by the two enzymes, the kinetic constants and the catalytic efficiencies of *Il*-DyP4 differed markedly from those of *Il*-MnP6. *Il*-DyP4 was able to oxidize Mn^2+^ with a *k*_*cat*_/*K*_*m*_ value only an order of magnitude lower than that of the *Il*-MnP6, but higher than that of *Pleos*-DyP1 and *Pleos*-DyP4 reported from *P. ostreatus* (Fernández-Fueyo et al. 2015). Remarkably, *Il*-DyP4 exhibited a lower *k*_*cat*_/*K*_*m*_ for VA, a nonphenolic lignin model compound, similar to that of native DyP from *I. lacteus* and both AjPs from *Auricularia auricula*-*judae*, showing a certain ligninolytic activity (Salvachúa et al. 2013; Linde et al. 2014). However, the VA-oxidizing activities of *Il*-MnP6 was not detected under our test conditions, this was different from a previous report which shows *Il*MnP1 and *Il*MnP2 from *I. lacteus* CD2 that are able to oxidize VA in the presence of Mn^2+^ (Qin et al. 2017). Qin et al. (2017) indicated that either malonate or oxalate buffer was essential in the VA degradation by the two MnPs. Therefore, the sodium tartrate buffer was changed to the malonate buffer in order to re-examine the ability of VA oxidation in *Il*-MnP6 from *I. lacteus* F17. Interestingly, the assay revealed the activity of *Il*-MnP6 for VA oxidation in 50 mM malonate buffer (pH 3.5), and that the activity had improved with the increase in enzyme concentration (data not shown). Such results supports the findings from Qin et al. (2017), who showed that the Mn^3+^-malonate complexes may mediate oxidation of VA through the action of radicals. Yoshida and Sugano reviewed the characteristics of DyP-type peroxidases and showed that the *k*_*cat*_/*K*_*m*_ of bacteria DyPs for anthraquinone compounds was 10^2^–10^5^ s^−1^ M^−1^, whereas the *k*_*cat*_/*K*_*m*_ of basidiomycota DyPs reached 10^6^–10^7^ s^−1^ M^−1^ (Yoshida and Sugano 2015). In the present study, the recombinant *Il*-DyP4 from *I. lacteus* F17 exhibited higher catalytic efficiency (4.0 × 10^7^ s^−1^ M^−1^) for RBlue 19 than did basidiomycota DyPs from *I. lacteus*, *A. auricula*-*judae*, and *P. ostreatus* (Salvachúa et al. 2013; Linde et al. 2014; Fernández-Fueyo et al. 2015). Compared with *Il*-MnP6, *Il*-DyP4 showed a lower affinity for azo dye RBlack 5, although its catalytic efficiency was almost two orders of magnitude higher than that of *Il*-MnP6, and was similar to that of native DyP from *I. lacteus* and superior to that of other DyPs (Salvachúa et al. 2013). Therefore, these results revealed the remarkable ability of *Il*-DyP4 to catalyze these substrates. These data also demonstrated that anthraquinone dye RBlue 19 and ABTS were the preferential substrates for *Il*-DyP4, which was consistent with the report by Linde et al. (2015). For *Il*-MnP6, Mn^2+^ was reconfirmed as its preferred substrate.

The effect of H_2_O_2_ and other compounds at different concentrations on the enzymatic activity of the two enzymes was also examined. Although, the catalytic efficiency of *Il*-DyP4 for H_2_O_2_ was an order of magnitude higher than that of *Il*-MnP6, it appeared to be more susceptible than *Il*-MnP6 at lower H_2_O_2_ concentrations (< 7.0 mM). This could be attributed to the different amino acid residues in the heme pocket influencing the enzymatic stability. Ogola et al. (2010) increased the H_2_O_2_ resistibility of a DyP-type peroxidase from *Anabaena* sp. by the site-directed mutagenesis of methionine residues. By contrast, reducing agents, such as DTT, thiourea, and β-mercaptoethanol (1 and 10 mM), significantly affected the enzymatic activity of *Il*-MnP6, resulting in the reduction of disulfide bonds and inactivation of the enzyme; by contrast, *Il*-DyP4 only contains a cysteine residue and lacks disulfide bonds, explaining the only small effect of this enzyme on these compounds. Interestingly, 1 and 10 mM of Fe^2+^ and Fe^3+^ caused a dramatic decrease in activities of both enzymes, compared with other metal ions. To the best of our knowledge, the reason for this inhibition is unclear. However, it has been known that the two types of heme peroxidase share the same catalytic cycle, using H_2_O_2_ as an electron acceptor to form two oxidized intermediates, compound I and II, which are then reduced back to the native enzymes. In both native proteins, heme iron (protoporphyrin IX) is a penta-coordinated high-spin state Fe^3+^, bound to the imidazole ring N of the conserved proximal histidine (His), which has been proposed to be a key amino acid residue. The distance between the heme iron and proximal His influences the redox potential of ligninolytic peroxidases (Martínez 2002). In our assay, the high concentration of exogenous Fe ions might have disturbed and altered the formation of covalent bonds between the heme iron and His, leading to enzyme inactivation. Exogenous irons might be able to interact with the imidazole groups at a high concentration, and affect the bonding state of the active center Fe^3+^ of the heme porphyrin ring. Thus, more detailed studies are needed to further characterize the inhibition mechanism of Fe ions on the activity of these two enzymes. Guo et al. (2009) proposed a possible inhibition mechanism of lanthanum ions on the activity of horseradish peroxidase (HRP). They found that La^3+^ can combine with amide groups of the polypeptide chain and destroy the native structure of HRP. Tayefi-Nasrabadi et al. (2006) also reported conformational changes and activity alterations induced by Ni^2+^ ions in HRP.

Differences were observed in the CD spectra between *Il*-DyP4 and *Il*-MnP6, both in terms of the α-helix and β-sheets contents, in agreement with the predicted molecular models. The UV–Vis absorption spectrum of *Il*-DyP4 had features similar to that of *Il*-MnP6. A characteristic CT band at 634 nm was observed in the spectrum of *Il*-DyP4, indicating a high-spin heme Fe^3+^ state. Also, the R_Z_ value was similar to that of native DyP from *I. lacteus* (Salvachúa et al. 2013), thus, it appears that the in vitro refolding conditions used in this study were optimized. A similar pattern was observed in *Il*-MnP6: a characteristic CT band at 632 nm was obvious, and the R_Z_ value (3.17) was almost identical to that native MnP from the white-rot fungus *P. chrysosporium* (Whitwam and Tien 1996). Hence, despite the tedious refolding process, *Il*-MnP6 was reconstituted successfully from inclusion bodies.

Further investigations of the midpoint redox potential of the two enzymes were carried out, and the values of $E^{m^{\prime}}_{\text{Fe}^{3+}/{\text{Fe}^{2+}}}$ indicated that *Il*-DyP4 was superior to *Il*-MnP6, particularly at pH 3.5. Nevertheless, the two enzymes were differentially affected by changes in pH. The impact of pH on the redox potential of *Il*-DyP4 was bigger than that of *Il*-MnP6 (Table 4), indicating that *Il*-DyP4 was more sensitive to changes in pH. Moffeta and colleagues indicated that the redox potential of heme peroxidases is influenced by several factors, including the electronic nature of the amino acids ligating the heme, and the electrostatic interactions with residues surrounding the heme, as well as the solvent accessibility of the heme (Moffeta et al. 2003).

To determine the potential application ability of the two enzymes, five different structures of 16 synthetic dyes were selected and a lower enzyme concentration of 25 nM was used to decolorize these dyes in the two same enzymatic reaction systems. The differences in decolorization percentage of 15 synthetic dyes were obvious between the two enzymes, except for Orange G. *Il*-DyP4 was superior to *Il*-MnP6 in all the structure types of dye tested and showed high oxidation capacities at pH values of 3.5–4.5, which were optimal pH values for most reported DyPs and MnPs (Hofrichter et al. 2010). This was suggested to be because of the increased redox potential of the oxidized heme at low pH (Petruccioli et al. 2009). Our voltammetric experiment data illustrated the strongest oxidization ability of *Il*-DyP4 at pH 3.5, and showed that the redox potential declined dramatically with the increase in pH. Thus, this result explained why DyPs required acidic pH conditions to complete substrate oxidization. In addition, in the case of the *Il*-DyP4 system, dye decolorization was carried out directly by the addition of enzyme and H_2_O_2_, whereas MnPs catalyzed the Mn^2+^-mediated oxidation reaction, and an optimal concentration of Mn^2+^ was required for the MnP decolorization system. Thus, the biotechnological application of MnPs would be limited because of the heavy metal contamination in treated industrial wastes, whereas DyPs are only robust under acidic conditions, which would not be appropriate in some industrial applications, such as the treatment of neutral and alkaline waste in pulping and bleaching. Therefore, there is still much research to be done to enhance the industrial applicability of these enzymes.

Poly R-478, a structurally complicated and recalcitrant anthraquinone derivative, is a suitable indicator of the ability of white-rot fungi to degrade lignin, and it was used as a model compound for the measurement of ligninolytic activity (Zhao et al. 2015). The results of *Il*-DyP4 to decolorize Poly R-478 highlighted its ligninolytic activity and its ability to oxidize lignin and related recalcitrant compounds. The precise structure of *Il*-DyP4 and its key roles in lignin degradation also need to be clarified in future studies.

## Additional file

**Additional file 1.** Additional tables and figures.

## Abbreviations

DyP: dye decolorizing peroxidase
MnP: manganese peroxidase
*I. lacteus* F17: *Irpex lacteus* F17
*E. coli*: *Escherichia coli*
Rz: Reinheitszahl
H_2_O_2_: hydrogen peroxide
GSSG: glutathione
DTT: dithiothreitol
DMP: 2,6-dimethoxyphenol
ABTS: 2,2′-azino-bis(3-ethylbenzothiazoline-6-sulfonic acid)
VA: veratryl alcohol
PMSF: phenylmethanesulfonyl fluoride
IPTG: isopropyl-β-d-thiogalactopyranoside
RBlue 19: reactive blue 19
RBlack 5: reactive black 5
EDTA: ethylene diamine tetraacetic acid
SDS–PAGE: sodium dodecyl sulfate–polyacrylamide gel electrophoresis
PGE: pyrolytic graphite electrode
CD: circular dichroism
