# Supplementary material for: Comprehensive investigation of a dye-decolorizing peroxidase and a manganese peroxidase from Irpex lacteus F17, a lignin-degrading basidiomycete
Source: AMB Express. 2018 Jul 17;8:119. doi: 10.1186/s13568-018-0648-6 (PMC6049852; doi:10.1186/s13568-018-0648-6)
Supplement: Supplementary file 1 — Additional file 1. Additional tables and figures. [file 13568_2018_648_MOESM1_ESM.docx]

**Additional Material**

**Comprehensive investigation of a dye-decolorizing peroxidase and a manganese peroxidase from *Irpex lacteus* F17, a lignin-degrading basidiomycete**

Zihong Duan^1,2^ **·** Rui Shen^1,2^ **·** Binjie Liu^1,2^ **·** Mengwei Yao^1,2^ **·** Rong Jia^1,2^

^1^School of Life Science, Economic and Technology Development Zone, Anhui University, 111 Jiulong Road, Hefei, Anhui 230601, People’s Republic of China

^2^Anhui Key Laboratory of Modern Biomanufacturing, Anhui University, Hefei 230601, People’s Republic of China

Zihong Duan and Rui Shen contributed equally to this work.

Corresponding author: Rong Jia. Tel.: +86 551 63861063

E-mail addresses: [ahdxjiarong@126.com](mailto:ahdxjiarong@126.com) (R.Jia)

**Table S1** Primers used in this study

| Primer | Nucleotide sequence |
| --- | --- |
| DyPf | ATGCAGCTCAGACACTTCCTC |
| DyPr | TCAAGCAGCAAATTTCTCGACAATAG |
| MnPf | ATGGCCTTCAAACAACTTGTC |
| MnPr | TTAGGATGGCGGAACAGG |
| NcoI-DyPf | ATACCATGGGCAGTGCCGATAATGATAGCCTG |
| XhoI-DyPr | GTGCTCGAGTGGCTGCAAATTTTTCCACAATTGC |
| NcoI-MnPf | ATACCATGGCGATCACAAAGCGGGTTGC |
| XhoI-MnPr | GTGCTCGAGGGATGGCGGAACAGGGGC |

**Table S2** Amino-acid sequence identities between the five DyPs from the *I. lacteus* F17 genome (NCBI protein references included). The number of the same residue pairs considered for each comparison is shown after the identity percentage. The selected enzyme was marked in red

**Table S3** Amino-acid sequence identities between the thirteen MnPs from the *I. lacteus* F17 genome (NCBI protein references included). The number of the same residue pairs considered for each comparison is shown after the identity percentage. The selected enzyme was marked in red

**Table S4** Eight peptides of the purified *Il-*DyP4 analyzed by LC-MS

| m/z | amino acid sequences |
| --- | --- |
| 2537.24 | MGSAGNDSLPFENIQGDILVGMKK |
| 2056.07, | DKEKFVFFHINNATAFK |
| 3012.45 | GTNVDGVFLIGSNDESITAQYRDDLNAK |
| 2422.23 | SKDPVMNRPSWALDGSFLVFR |
| 2774.4 | YVLDNALQNQAGNLTVEEGAELLGSR |
| 2628.26 | WKSGAPIDLSPDFDDPALGNDIER |
| 2397.29 | FPFSKGPSIQLGLDPVIGQGSPR |
| 1372.64 | FAALEHHHHHH |

**Table S5** Seven peptides of the purified *Il*-MnP6 analyzed by LC-MS

| m/z | amino acid sequences |
| --- | --- |
| 902.45 | RVACPDGK |
| 2581.09, | DDIQENLFDGGQCGEEVHESFR |
| 2582.26 | HNITPGDFIQFAGAVGVSNCPGAPR |
| 2384.23 | GTLFPGTAGNQGEVQSPLRGEIR |
| 1167.65 | KLSILGHDER |
| 2188.12 | VKPATFPAGLSNKDVEQAC |
| 3297.62 | ATPFPTLPTDPGPSTSVAPVPPSLEHHHHHH |

*Il*-DyP1 ---------MRTV---ALYASLLSLLACTCAAAGEHGQ-VGHRHIKRLARKAPLLAAFSG 47

*Il*-DyP2 MACIVERKPLSFVDATPQTEALKNFLAATAAFVAVSQ—PAFAYHVKRARTTSLLGSFPG 58

*Il*-DyP3 ---------MRFS---GLLVASLSFIAHICTVSGAHVSAVKDELTKRDTRSAPLLASFPG 48

*Il*-DyP4 -------------------MQLRHFLAATAAFSAISQ—SSLAYHVKRARSTPLIGSFPG 38

*Il*-DyP5 -------------------MQLKHFLAATAAFSAVTQ—SAFAYHVKRARSSPLIGSFPG 38

::* .: . : :*.: *:.:* *

*Il*-DyP1 HGALPTLQHILNIDVTRGSMLPIKNIQGDIL------VGMKKNKERFVFFHINDASRFKD 101

*Il*-DyP2 QGALPTVAQVQSTSSG-NDSLPLENIQGDIL------VGMKKQKEQFVFFHINNATTFKK 111

*Il*-DyP3 QGLLPTLEQILTFNATNGTFLPIQNIQGDILPLAVSSVGMKKQKERFVFFHINDPTRFKG 108

*Il*-DyP4 QPPLPTIAQVQSTSAG-NDSLPFENIQGDIL------VGMKKDKEKFVFFHINNATAFKS 92

*Il*-DyP5 QPPLPTIAEVQSSSAG-NDSLPFENIQGDIL------VGMKKDKEKFVFFHINNATTFKS 92

: ***: .: . . . **::******* *****:**:*******: : **

*Il*-DyP1 VLKTYAPANITSVATLVSPAPLQPLAFVNVAFSRTGLSTLGVKDNLGDNAFNAGQFADAE 161

*Il*-DyP2 VLKTYAPANITSIQTLLSAPSAQPLAFVNLAFSQSGATALGVTDDLGDSFFSAGQFADAS 171

*Il*-DyP3 VLKTYASANITSVATLVSPPSSQPLAFVNVAFSQTGLTTLGVSDNLGDTQFASGQFADAS 168

*Il*-DyP4 VLKTYAPANITSVATIIGPVANQPLAFVNLAFSHAGFGALNVTDDLQDTAFSDGQFKDSP 152

*Il*-DyP5 FLKTYAPANITSVQTIIGPASGQPQAFVNLAFSHTGFGALGVADDLQDTAFTAGQFADAP 152

.***** *****: *::. ** ****:***::* :*.* *:* *. * *** *:

*Il*-DyP1 GLGDDTGVWESAFKGTHIHGVFLIGSDQDEFLQTYTHDLNALFGSSLSVEYTLDAAARPG 221

*Il*-DyP2 ALGDDTSNWDSTFTGTDVHGVFLIGSDETSFVEQYQSDLEAKLGDAWTVSLTIASAARPG 231

*Il*-DyP3 GLGDDINKWETAFKGTNIHGVFLIGSDQDSYLTAYNNDLNELFGSSLSIVYTLNSAARPG 228

*Il*-DyP4 NLGDDTSTWEEAFKGTNVDGVFLIGSNDESITAQYRDDLNAKFGDAWTIVYDLDSAARPG 212

*Il*-DyP5 SFGDDTSTWEEAFKGTNVDGVFLIGSDDVTTTNQYRDDLKAKLGDAWTVLLDLDSAARPG 212

:*** . *: :*.**.:.*******:: * **: :*.: :: : :*****

*Il*-DyP1 PEAGHEHFGFLDGISNPAVTGFDSPSPGQSLVPAGVILTGHTGDT-TPRPRWAKDGSFMV 280

*Il*-DyP2 DEAGHEHFGYLDGISNPTITGFGTALPGQSVVDPGIILAGRTGDTV-SRPSWALDGSFLV 290

*Il*-DyP3 AQAGHEHFGFLDGISNPAVIGFNDPLPGQTLALPGIILTGRLGDGTFTRPSWTKDGSFLV 288

*Il*-DyP4 NEKGHEHFGYLDGISNPTIPGFGTPHPGQAVVDPGIIFTGRSKDPVMNRPSWALDGSFLV 272

*Il*-DyP5 AEKGHEHFGYLDGISNPTIPGFGTPFPGQAVVDSGVIFAGRTNDPVTNRPSWALDGSFLV 272

: ******:*******:: **. ***::. *:*::*: * ** *: ****:*

*Il*-DyP1 FRKLKQLVPEFRKWTLDNAIQNKAGNLTVEEGAEYLGARMMGRWKSGAPIDIASEDDDPA 340

*Il*-DyP2 FRKLKQLVPEFNKWTLANAIQNASGNLTAQEGADLLGSRMFGRWKSGAPIDLSPDVDDAS 350

*Il*-DyP3 FRKLKQLVPEFNKWTIDNAVQNKAGTLTVQEGAEFIGARMIGRWKSGAPLDIVPETDDST 348

*Il*-DyP4 FRKLKQLVPEFNKYVLDNALQNQAGNLTVEEGAELLGSRMFGRWKSGAPIDLSPDFDDPA 332

*Il*-DyP5 FRKLKQLVPEFHKWTLDNALQNQAGNLTVEEGALLLGSRMFGRWNSGAPIDLTPDVDDPT 332

***********.*:.: **:** :*.**.:*** :*:**:***:****:*: : ** :

*Il*-DyP1 LGADPHRNNNFNYAHPGSSLASDQTHCPFSAHTRKLNPRADQA—GTDTPNHAIRAGTPY 398

*Il*-DyP2 LGTDPQRNNNFNYTHAGSDITSDESRCPFSAHIRKTNPRDLES—VIGHANHAIRAGTPY 408

*Il*-DyP3 LGADPQRNNKFDFAHLTSNLISDQTYCPFSAHIRKTNPRADLA—DANTINHAIRAGTPY 406

*Il*-DyP4 LGNDIERNNNFNYSHPGSDLATDQTRCPFTAHIRKTNPRDLEGQGLFGDTFHAIRAGTPY 392

*Il*-DyP5 LGNDPQRNNDFNYIHPGEDLTTDETRCPFTAHVRKTNPRDLEAQGLIPDLFHAIRAGTPY 392

** * .***.*:: * ..: :*:: ***:** ** *** . *********

*Il*-DyP1 GAELSDVEAASNTSQIDRGLAFVMYQSNIGNGFQFQQLAWANTENFPPFKNV—TPGIEP 456

*Il*-DyP2 GPELTDDESSSGTTSTDRGLAFVEYQSNIGNGFRTQQEVWANAAAFPFGKTE—SIGLDP 466

*Il*-DyP3 GPEVSAAEAHANTTQTDRGLAFVEYQSSIANGFRFQQVNWANTANFPPLKSV—NAGFDP 464

*Il*-DyP4 GPEVTDYEASSNTTTIDRGLAFVEYQSVIGNGFRFQQQAWANNPRFPFSKGPSIQLGLDP 452

*Il*-DyP5 GPEVTDAESNSNTTSIDRGLAFVEYQSVISNGFRFQQLNWANNANFPFNKSE—PLGLDP 450

* *:: *: :.*: ******* *** *.***: ** *** ** * *::*

*Il*-DyP1 IIGRGSPRVATGFDPHDQAKTYTMPDFVVSNGGEYFFVPSISAISDIIAA 506

*Il*-DyP2 VIGQGD-RTTYGLNAQNVTESYSVPSFVISNGGEYFFSPSITALVETFAA 515

*Il*-DyP3 VIGRGTPRTAVGLDPNDQTKSYTLPDFVVSNGGEYFFSPSISAITGTIAA 514

*Il*-DyP4 VIGQGSPRETFGLDPRNASESFTVPQVIISNGGEYFFSPSITAIVEKFAA 502

*Il*-DyP5 VIGQGT-RQTFGLDPRNASDSLTIPQIIISNGGEYFFSPSITALVEHFGA 499

:**:* * : *:: .: :.: ::*..::******** ***:*: :.*

**Fig. S1** Amino acid sequence alignments of five DyPs from *I. lacteus* F17 genome with Clustal X. Predicted N-terminal signal peptide sequences prior to the mature proteins are colored *orange*. Highlighted residues include the following: (i) the red box showed the GXXDG motif, conserved aspartic acid were shown in *red*; (ii) proximal histidine residues were shown in *pink*, distal arginine residues were shown in *green*; (iii) radical-forming residues: tryptophans were shown in *purple*, tyrosines were shown in *blue*; (iv) cysteines were shown in *cyan*. *Symbols below the sequences* represented full conservation of the same (*asterisk*) or equivalent residues (*colon*) and partial conservative residues (*dot*).

*Il*-MnP1 --------------------------------------MAFKHLIAALSIVLSFGIAQ-- 20

*Il*-MnP2 --------------------------------------MAFKTILAFVTLATAALAAP— 20

*Il*-MnP3 --------------------------------------MAFKTLFALATLATAAFA---- 18

*Il*-MnP4 --------------------------------------MAFQSLFTLVALAAAVVAVPAP 22

*Il*-MnP5 MDGGEDEESSVLLHISSSTLTSPLSSLTAFQTTTITVDMAFKALVALASLATAVFAAPAA 60

*Il*-MnP6 --------------------------------------MAFKQLVTALSIALSFSVAQ-- 20

*Il*-MnP7 ------------------------------------------------------------ 0

*Il*-MnP8 --------------------------------------MAFKQLVAALTVALSLGVAQ-- 20

*Il*-MnP9 --------------------------------------MAFKQLVTALSIALSFSVAQ-- 20

*Il*-MnP10 --------------------------------------MAFKQLAAALSLAL---LAH-- 17

*Il*-MnP11 --------------------------------------MAFKQLVAALTVALSLGVAQ-- 20

*Il*-MnP12 --------------------------------------MAFKQLAAALSLAL---LAH-- 17

*Il*-MnP13 --------------MQAAFMHGLRGRYKSTAEEHGNGNMAFKALLALLTVTSAVLAAPQD 46

*Il*-MnP1 AAITKRVACPDGKNTATNAACCSLFAIRDDIQANLFDGGECGEEVHESFRLTFHDAIGT- 79

*Il*-MnP2 ---SSRVTCSPGR-VVGNGACCKWFDVLDDIQENLFDGGVCGEEVHESLRLTFHDAIGFS 76

*Il*-MnP3 -APSPLVSCGGGR-SVKNAACCAFFPVLDDIQANLFSGGTCEEEAHEAIRLIFHDAIGFS 76

*Il*-MnP4 --QDAQVNCGGGR-FVKNAACCAWFPVLDDIQENLFSGSLCAEEAHEALRLTFHDAVGFS 79

*Il*-MnP5 PAPSPLVSCGNGR-SVQNAACCAWFPVLDDIQANLFSGGTCEEEAHEAIRLTFHDAIGFS 119

*Il*-MnP6 AAITKRVACPDGKNTATNAACCALFAIRDDIQENLFDGGQCGEEVHESFRLTFHDAIGI- 79

*Il*-MnP7 -----------------------------------------------------MFYVGFS 7

*Il*-MnP8 GAITRRVACPDGVNTATNAACCSLFAIRDDIQQNLFDGGECGEEVHESFRLTFHDAIGI- 79

*Il*-MnP9 AAITKRVACPDGKNTATNAACCALFAIRDDIQENLFDGGQCGEEVHESFRLTFHDAIGI- 79

*Il*-MnP10 GAVVRRVTCPDGVNTATNAACCSLFAVRDDIQQNLFDNGQCGEDVHESFRLSFHDAIGIS 77

*Il*-MnP11 GAITRRVACPDGVNTATNAACCSLFAIRDDIQQNLFDGGECGEEVHESFRLTFHDAIGI- 79

*Il*-MnP12 GAVVRRVTCPDGVNTATNAACCSLFAVRDDIQQNLFDNGQCGEDVHESFRLSFHDAIGIS 77

*Il*-MnP13 VTAANKVSCGGGR-VAGHAQCCKWYDVLDDIQKNLFDGGECGEEVHESLRLTFHDAIGFS 105

:*

*Il*-MnP1 ------GSFGGGGADGSIIVFDDIETNFHANNGVDEIIDEQKPFIARHNITPGDFIQFAG 133

*Il*-MnP2 LSAEREGKFGGGGADGSIMAFAEIETNFHANNGVDEIVEAQRPFAIKHKVSFGDFIQFAG 136

*Il*-MnP3 NSLTQQGKFGGGGADGSILAFSDIETSFAANFGLDFTTEAFIPFALAHGVSFGDFVHFAG 136

*Il*-MnP4 IAAEREGQFGGGGADGSILAFSDIETSFAANFGLDFTTEAFIPFALAHKVSFGDFVQFAG 139

*Il*-MnP5 QALTKEGKFGGGGADGSILAFSDIETNFAANFGLDFTTEAFIPFAVAHQVSFGDFVQFAG 179

*Il*-MnP6 ------GSNGGGGADGSIAVFEAIETAFHANNGVDEIIDEQKPFIARHNITPGDFIQFAG 133

*Il*-MnP7 IKANLEGKFGGGGADGSILAFSDIETSFAANFGLDFTTEAFIPFALAHNVSFGDFVQFAG 67

*Il*-MnP8 ------GSNGGGGADGSIAVFEDIETAFHANNGVDEIIDEQKPFLARHNITPGDFIQFAG 123

*Il*-MnP9 ------GSNGGGGADGSISVFEDIETAFHANNGVDEIIDEQKPFVARHNITPGDFIQFAG 153

*Il*-MnP10 PKIAATGQFGGGGADGSIILFEEIETNFHANIGVDEIVDEQKPFIARHNITPGDFIQFAA 137

*Il*-MnP11 ------GSNGGGGADGSIAVFEDIETAFHANNGVDEIIDEQKPFLARHNITPGDFIQFAG 133

*Il*-MnP12 PKIAATGQFGGGGADGSIILFEEIETNFHANIGVDEIVDEQKPFIARHNITPGDFIQFAA 137

*Il*-MnP13 LSAQREGKFGGGGADGSIMAFAEIETKFHANNGVDEIIEAQRPFALNHSVSFGDFIQFAG 165

*. ********* * *** * ** *:* : ** * :: ***::**.

*Il*-MnP1 AVGVSNCPGAPRLDFFLGRPNPVAAAPDKTVPEPFDTVDSILARFKDAGGFTPAEVVALL 193

*Il*-MnP2 AVGVSNCLGGPRLEFMAGRSNISRAAPDLTVPEPSDSVDKILARMGDAG-FSSSEVVDLL 195

*Il*-MnP3 AVAASNCLGGPRLQFMAGRPNNSRASPDGLVPQPTDGVDKIFARMADAG-FSPVELVHLL 195

*Il*-MnP4 AVGVSNCIGGPRLQFLAGRSNNSRPSPDNLVPEPTDSAEKIFERLQDIG-FSPIEVVHLL 198

*Il*-MnP5 AVGASNCLGGPRLQFLAGRPNNSQPSPDGLVPEPTDSADKIFARMQDAT-FSPTELVHLL 238

*Il*-MnP6 AVGVSNCPGAPRLDFFLGRPNPVAPAPDKTVPEPFDSVTSILARFKDAGNFSPEEVVALL 193

*Il*-MnP7 AVGVSNCIGGPRLKFLAGRSNISQPSPDGLVPEPTDSADKIFDRLADIG-FSPIEVADLL 126

*Il*-MnP8 AVGVSNCPGAPRLDFFLGRPNPVAPAPDKTVPEPFDTVDSILARFADAGGFSPAEVVALL 173

*Il*-MnP9 AVGVSNCPGAPRLDFFLGRPNPVAPAPDKTVPEPFDSVTSILARFKDAGNFSPEEVVALL 213

*Il*-MnP10 AVGVSNCPGAPRLDFFLGRPAATQPAPDKTVPEPFDTVDTILERFADAGNFTPAEVVALL 197

*Il*-MnP11 AVGVSNCPGAPRLDFFLGRP----PAPDKTVPEPFDTVDSILARFADAGGFSPAEVVALL 189

*Il*-MnP12 AVGVSNCPGAPRLDFFLGRPAATQPAPDKTVPEPFDTVDTILERFADAGNFTAAEVVALL 197

*Il*-MnP13 AVGVSNCGGGPRLQFLAGRSNSSKAAPDGTVPEPFDSTDKILARMGDAG-FSPSEVVDLL 224

**..*** *.***.*: ** :** **:* * . .*: *: * *: *:. **

*Il*-MnP1 GSHTIAAADHVDPTIPGTPFDSTPEVFDTQVFVE------------------VQLRGTLF 235

*Il*-MnP2 ISHTVAAQDHVDPTIPGTPFDSTPSEFDPQFFVEVSFNHAIVKRSDLRVLLQTLLKGTLF 255

*Il*-MnP3 TAHTVSAQYEVDTDVAGSPFDSTPSAFDNQFFVE------------------SLLHGTAF 237

*Il*-MnP4 TAHTVSAQYEVDTDVAGSPFDSTPSSFDNQFFVE------------------SLLKGTAF 240

*Il*-MnP5 AAHTVAAQYEVDTDVAGSPFDSTSSSFDTQFFVE------------------SLLTGTAF 280

*Il*-MnP6 GSHTIAAADHVDPTIPGTPFDSTPEVFDTQVFLE------------------VQLRGTLF 235

*Il*-MnP7 AAHSVSAQYEVDTDVAGSPFDSTPDTFDTQFFVE------------------SLLKGTAF 168

*Il*-MnP8 GSHTIAAADHVDPTIPGTPFDSTPEVFDTQVFLE------------------VQLRGTLF 215

*Il*-MnP9 GSHTIAAADHVDPTIPGTPFDSTPEVFDTQVFLE------------------VQLRGTLF 255

*Il*-MnP10 VSHTIAAADEVDPTIPGTPFDSTPEVFDSQFFVE------------------TQLRGTGF 239

*Il*-MnP11 GSHTIAAADHVDPTIPGTPFDSTPEVFDTQVFLE------------------VQLRGTLF 231

*Il*-MnP12 VSHTIAAADEVDPTIPGTPFDSTPEVFDSQFFVE------------------TQLRGTGF 239

*Il*-MnP13 ASHSVAAQDHVDASIPGTPFDSTPSTFDAQFFVE------------------TLLKGTLF 266

:*:::* .** : *:***** . ** *.*:* * ** *

*Il*-MnP1 PG--TGGNQGEVQSPLRGEIRLQSDHDLARDSRTACEWQSFVNNQAKLQSAFKAAFKKLS 293

*Il*-MnP2 PG—NGSNVGELQSPLRGEFRLQSDALLARDPRTACEWQSFVNNQRLMVTKFEAVMSKLA 313

*Il*-MnP3 TG---NGKGGESMSPIPGEFRLASDFVISRDSRAACEWQALATDHQAMVNNFQAAMSKLS 294

*Il*-MnP4 TG---NGQGGEVTSPIPGEFRLQSDFAISRDSRTACEWQSLVTNHANMVSKFETVMAKLA 297

*Il*-MnP5 TG---NGQGGEAMSPIPGEFRLASDFVISRDQRAACEWQSLVNDHQTMVNNFEAAMAKMA 337

*Il*-MnP6 PG--TAGNQGEVQSPLRGEIRLQSDHDLARDQRTACEWQSFVNNQAKLQRNFKAAFKKLS 293

*Il*-MnP7 TG---NGEGGEVMSPIPGEFRLQSDFAISRDSRAACEWQSFVNNHNAMVTKFETVMSKLA 225

*Il*-MnP8 PGIRTGGNQGEVESPLRGEIRLQSDHDLARDSRTACEWQSFVNNQVKLQTAFKAAFKKLA 295

*Il*-MnP9 PG--TAGNQGEVQSPLRGEIRLQSDHDLARDQRTACEWQSFVNNQAKLQRNFKAAFKKLS 293

*Il*-MnP10 PG—TAGNQGEVESPLAGELRLQSDSELARDARTACEWQSFVGNQQKIQTAFKAAFQKMA 297

*Il*-MnP11 PGIRTGGNQGEVESPLRGEIRLQSDHDLARDSRTACEWQSFVNNQVKLQTAFKAAFKKLA 299

*Il*-MnP12 PG--TAGNQGEVESPLAGELRLQSDSELARDARTACEWQSFVGNQQKIQTAFKAAFQKMA 297

*Il*-MnP13 PG--NGSNQGEVQSPLHGEFRLQSDFELARDPRTACEWQSFITDHSSMVRKFEAAMAKLA 324

* .: ** **: **:** ** ::** *:*****:: :: : *::.: *::

*Il*-MnP1 VLGHNINNLIDCSEVIPEPPNVKVKPATFPAGITHADVE-QACATTPFPTLATDPGPATS 352

*Il*-MnP2 VLGHNPRDLVDCSEVIPVPPRAKTNVAVLPAGKTRADVQ-AACAATPFPTLQTAPGPATS 372

*Il*-MnP3 TLGQTGNNLIDCSDVIPVPIAAKFT-ATLPPGKTMADIDRSGCASFPFPSLATQPGPVTS 353

*Il*-MnP4 TVGQNPNNLIDCSDVIPVPPAAKVTTGSFPPGKSKADVQ-SACAATPFPNLATQPGPVTS 356

*Il*-MnP5 ILGQTASELIDCSDVIPVPASAKFT-ATFPPSKSIKDVN-SACSAFPFPSLATQPGPVTS 295

*Il*-MnP6 ILGHDERRLIDCSEVIPEPPNVKVKPATFPAGLSNKDVE-QACRATPFPTLPTDPGPSTS 352

*Il*-MnP7 TVGQDPSNLVDCSDVIPVPPAAKVQVGHLPPGKTLDDVD-SACAATPFPSLATQPGPVTS 284

*Il*-MnP8 VLGHDINNMVDCSEVIPEPPNVKIKAATFPAGQTNADVE-QACASTPFPTLATDPGPATS 354

*Il*-MnP9 ILGHDERRLIDCSEVIPEPPNVKVKPATFPAGLSNKDVE-QACRATPFPTLPTDPGPSTS 352

*Il*-MnP10 VLGVDTSKMVDCSELIPVPPELKITAAHFPAGKTNADVE-QACASTPFPTLSTDPGPATS 356

*Il*-MnP11 VLGHDINNMVDCSEVIPEPPNVKIKAATFPAGQTNADVE-QACASTPFPTLATDPGPATS 350

*Il*-MnP12 VLGVDTSKMVDCSELIPVPPELKITAAHFPAGKTNADVE-QACASTPFPTLSTDPGPATS 356

*Il*-MnP13 VLGHDPRTLIDCSDVIPQPKGAKSNVAVLPAGKHRADIQ-ASCHQTPFPTLKTAPGPETS 383

:* ::***::** * * . :* . *:: .* ***.* * *** **

*Il*-MnP1 VAPVPPS-- 359

*Il*-MnP2 IAPVPPS— 379

*Il*-MnP3 VLPVTA--- 359

*Il*-MnP4 VLPVTA--- 362

*Il*-MnP5 VLPVTA--- 401

*Il*-MnP6 VAPVPPS-- 359

*Il*-MnP7 VLPVTA--- 290

*Il*-MnP8 VAPVPPS-- 361

*Il*-MnP9 VAPVPPS-- 359

*Il*-MnP10 VAPVPPS-- 363

*Il*-MnP11 VAPVPPS-- 357

*Il*-MnP12 VAPV----- 360

*Il*-MnP13 IPPVPPS-- 390

: **

**Fig. S2** Amino acid sequence alignments of thirteen MnPs from *I. lacteus* F17 genome with Clustal X. Predicted N-terminal signal peptide sequences prior to the mature proteins are colored *orange*. Highlighted residues include the following: (i) three conserved residues of Mn^2+^ binding sites were shown in *green*; (ii) three conserved residues in heme pockets were shown in *yellow*; (iii) Ca^2+^ binding sites were shown in *pink*; (iv) radical forming residues: tryptophans were shown in *red*, tyrosines were shown in *blue*; (v) cysteines were shown in *cyan*. *Symbols below the sequences* represented full conservation of the same (*asterisk*) or equivalent residues (*colon*) and partial conservative residues (*dot*).

**a** **b**


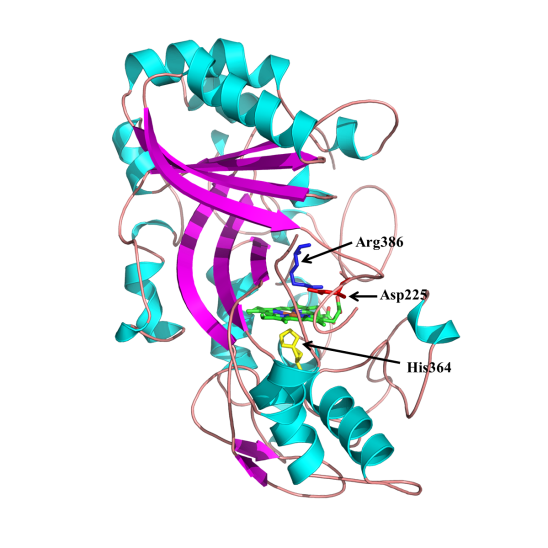

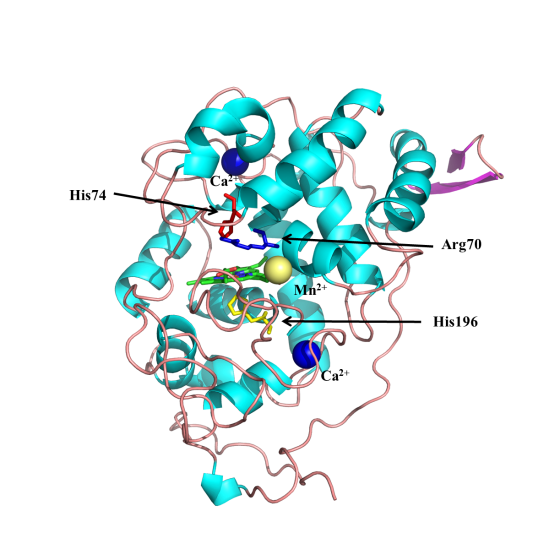


**Fig. S3** Molecular models of *Il*-DyP4 and *Il*-MnP6 by PyMOL software. **(a)** A model constructed using a typical class V DyP (PDB 3afv) as a template. **(b)** A model constructed using a typical MnP (PDB 2boq) as a template. The vital amino acid residues are indicated by the arrow.


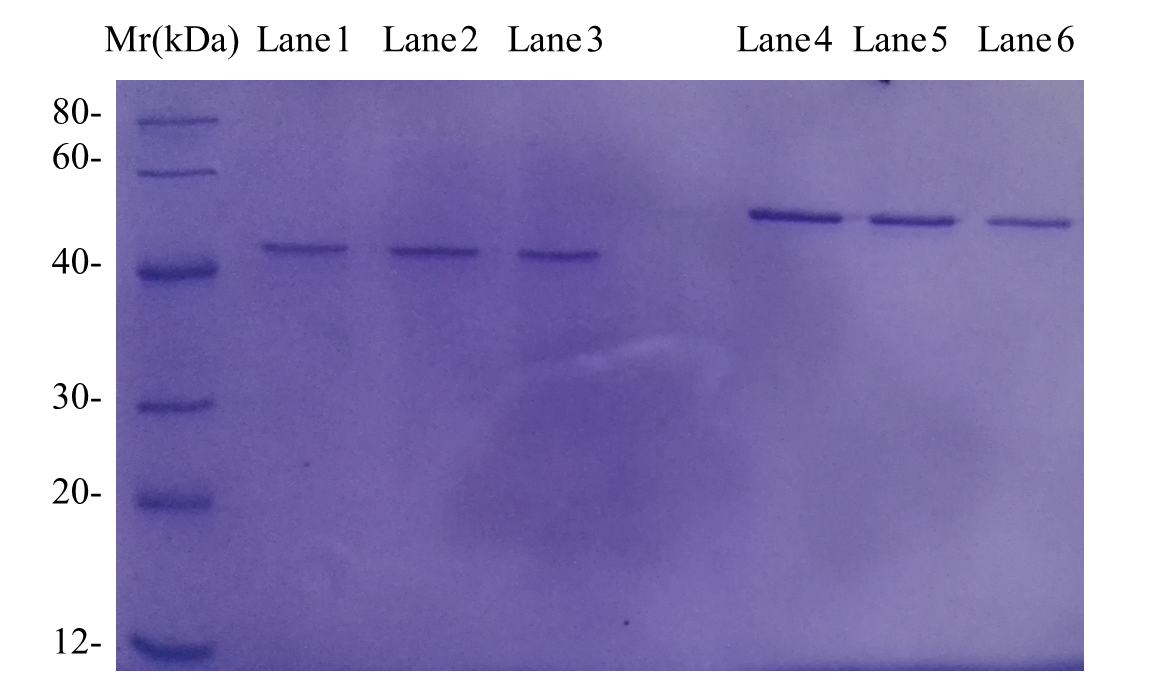


**Fig. S4** SDS-PAGE of recombinant *Il*-DyP4 and *Il*-MnP6 purified by a Ni-NTA column. Lane M: protein standards; Lane 1, 2 and 3: purified refolded *Il*-MnP6; Lane 4, 5 and 6: purified refolded *Il*-DyP4.





**Fig. S5** The far-UV CD of *Il*-DyP4 and *Il*-MnP6. Protein concentration was 0.1 mg mL^–1^ in 0.15 M phosphate buffer at pH 6.5.





**Fig. S6** Poly R-478 decolorization by *Il*-DyP4 and *Il*-MnP6. Experiments were conducted in 1.0 mL reaction mixtures containing 0.1 M sodium tartrate buffer (pH 3.5, 4.0, 4.5), 0.01% Poly R-478, 0.2 mM H_2_O_2_, and 100 nM enzyme. In addition, 1.0 mM of MnSO_4_ was included in reaction system of *Il*-MnP6.
